# Exploring the Potential of a Digital Intervention to Enhance Couple Relationships (the Paired App): Mixed Methods Evaluation

**DOI:** 10.2196/55433

**Published:** 2025-04-14

**Authors:** Catherine Aicken, Jacqui Gabb, Salvatore Di Martino, Tom Witney, Mathijs Lucassen

**Affiliations:** 1 School of Education, Sport and Health Sciences University of Brighton Brighton United Kingdom; 2 Faculty of Arts & Social Sciences The Open University Milton Keynes United Kingdom; 3 School of Social Sciences University of Bradford Bradford United Kingdom; 4 Institute of Epidemiology and Health Care University College London London United Kingdom; 5 School of Health and Medical Sciences City St George's, University of London London United Kingdom

**Keywords:** digital intervention, couple relationships, romantic relationships, relationship quality, app, digital technology, couples, internet-based intervention, evaluation research, mobile phone

## Abstract

**Background:**

Despite the effects of poor relationship quality on individuals’, couples’, and families’ well-being, help seeking often does not occur until problems arise. Digital interventions may lower barriers to engagement with preventive relationship care. The *Paired* app, launched in October 2020, aims to strengthen and enhance couple relationships. It provides daily questions, quizzes, tips, and detailed content and facilitates in-app sharing of question and quiz responses and tagged content between partners.

**Objective:**

To explore the potential of mobile health to benefit couple relationships and how it may do this, we examined (1) *Paired’s* impact on relationship quality and (2) its mechanisms of action.

**Methods:**

This mixed methods evaluation invited *Paired* subscribers to complete (1) brief longitudinal surveys over 3 months (n=440), (2) a 30-item web-based survey (n=745), and (3) in-depth interviews (n=20). For objective 1, survey results were triangulated to determine associations between relationship quality measures and the duration and frequency of *Paired* use, and qualitative data were integrated to provide explanatory depth. For objective 2, mechanisms of action were explored using a dominant qualitative approach.

**Results:**

Relationship quality improved with increasing duration and frequency of *Paired* use. Web-based survey data indicate that the Multidimensional Quality of Relationship Scale score (representing relationship quality on a 0-10 scale) was 35.5% higher (95% CI 31.1%-43.7%; *P*=.002), at 7.03, among people who had used *Paired* for >3 months compared to 5.19 among new users (≤1 wk use of *Paired*), a trend supported by the longitudinal data. Of those who had used *Paired* for >1 month, 64.3% (330/513) agreed that their relationship felt stronger since using the app (95% CI 60.2%-68.4%), with no or minimal demographic differences. Regarding the app’s mechanisms of action, interview accounts demonstrated how it prompted and habituated meaningful communication between partners, both within and outside the app. Couples made regular times in their day to discuss the topics *Paired* raised. Daily questions were sometimes lighthearted and sometimes concerned topics that couples might find challenging to discuss (eg, money management). Interviewees valued the combination of fun and seriousness. It was easier to discuss challenging topics when they were raised by the “neutral” app, rather than during stressful circumstances or when broached by 1 partner. Engagement seemed to be enhanced by users’ experience of relationship benefits and by the app’s design.

**Conclusions:**

This study demonstrates proof of concept, showing that *Paired* may have the potential to improve relationship quality over a relatively short time frame. Positive relationship practices became embedded within couples’ daily routines, suggesting that relationship quality improvements might be sustained. Digital interventions can play an important role in the relationship care ecosystem. The mixed methods design enabled triangulation and integration, strengthening our findings. However, app users were self-selecting, and methodological choices impact our findings’ generalizability.

## Introduction

### Background

Due to its prevalence and impacts, poor relationship health has been identified as a public health issue and deterioration of couple relationships as an epidemic [1]. Poor relationship quality (and similar constructs; eg, marital strain) negatively affects individual partners’ well-being [2,3], mental health [4,5], and physical health [6,7]; moreover, children are negatively impacted by poor parental relationship quality [8], conflict [9,10], and divorce [11,12]. Although an overwhelming majority of people worldwide ever marry [13], relationship distress and breakdowns are common. Approximately 50% of marriages in the United States and >40% of marriages in the United Kingdom end in divorce [14,15]; in addition, in the United Kingdom, 18% of ongoing couple relationships are estimated to be distressed [16].

Despite the importance of couple relationships, help seeking for relationship problems is often delayed or inadequate. Among people who are currently in a relationship, half of US adults, as well as a higher proportion of UK adults, do not seek advice for relationship issues from any source (not even web-based searches), and among those who do, the most common sources are friends and family [17]. Rigorously evaluated interventions such as couple therapy [18,19] and couple relationship education [20] (terms we use to include marital therapy and marital relationship education, respectively) are underused compared to less evaluated sources of advice (eg, self-help books; or talking with family, friends, or religious leaders) [21]. Possible reasons include accessibility, cost, social stigma, and the need for both partners’ simultaneous participation [22,23]. Couple relationship education is preventive, seeking to promote healthy relationships; yet, it often has a religious basis, conventionally focusing on marriage [24]. There is an opportunity for relationship care interventions that are independent of faith-based organizations and inclusive of diverse relationships.

### Potential for Digital Interventions in Relationship Care

Digital interventions may increase the accessibility of relationship care, their use fits with contemporary couple behaviors, and they may be effective in supporting behavior change. Taking these points in turn, compared to face-to-face interventions, mobile health (mHealth) interventions can be delivered discreetly, which is appropriate for sensitive or stigmatized matters, and they may further increase access by overcoming barriers such as inconvenience and cost [25]. Communications technology is embedded in many areas of contemporary coupledom: meeting romantic and sexual partners on the web is common [26-29], and information and communications technologies are used to complement couples’ in-person communication, sustain intimacy while apart, and end relationships [30]. Digital interventions can facilitate self-monitoring, which raises awareness and thus facilitates self-management of users’ behavior and emotions [31], and through cues, routines, and rewards, apps may be effective in habit formation within relationships [32].

Although digital interventions can be effective for supporting behavior change across diverse health areas [33-36], disengagement is generally high [37,38], and app quality is often poor. Many of the >250 new mHealth apps that are available daily [39] are not based on robust evidence or theory and may contain inaccurate or harmful information [40,41]. Empirical evidence on digital interventions for couples lags behind the emergence of new apps [42]. A recent review [42] found that most digital couple interventions were treatment focused (eg, for relationally distressed couples or for prevention of intimate partner violence) or lacked clarity about whether they were treatment focused or for primary prevention (ie, positive relationship care), making it unclear who should use them. The digital couple intervention that was most clearly focused on primary prevention consisted of a simple 1-time relationship assessment, while others were modular courses for couples to complete together, often linked to professional or coaching support [42]. Although human support may enhance engagement, these “blended” and structured interventions may negate some of the accessibility, convenience, and scalability advantages that mHealth can offer. We offer an evaluation of a fully digital and flexible intervention.

### The Paired App

*Paired* is a commercially available mobile app, launched in October 2020 and designed to help couples enhance their relationships. Its intended users are couples at any stage in their relationship, including same- and opposite-sex couples. It is not intended for couples experiencing relationship distress, who may require intensive intervention, and there is no human input targeted at the individual or couple (ie, it is not a blended or guided intervention). It is not based on any single theory of how relationships are sustained or improved but was developed with input from relationship science experts and informed by findings from the *Enduring Love?* study [43]. This study built upon practices theorizing—notably family practices [44] and practices of intimacy [45]—to examine the ways that daily interactions generate relationship quality, developing the concept of everyday relationship work [43]. The findings highlighted the importance of daily gestures and relationship work in the maintenance of long-term couple relationships [43]. During our study, *Paired* was available for free, in English, and in multiple countries, with most users in the United States and the United Kingdom. A paid-for version provides access to additional content and features. New content is continually added, and there is no defined course or set sequence of activities to complete. The app’s main features and functionality—described in the next subsection—did not change during data collection (and were common to free and paid-for versions). Therefore, this study evaluates an early version of *Paired* within the first 3 months of its launch.

*Paired* provides daily questions and weekly quizzes, intended to prompt couples to have frequent, open conversations on diverse topics. If individuals link their app account with their partner’s, question and quiz responses become mutually available when both partners have responded. Users can reply to their partner within the app and receive tips and links to preprepared topical content, including from therapists and academics specializing on couple relationships (which are searchable and accessible at any time). *Paired* can also be used independently: a user can access content and discuss questions with a partner who does not have the app.

### This Study

#### Approach and Theoretical Basis

Digital health interventions are complex interventions, operating within complex systems [46]. *Paired* has several dimensions of complexity; for example, users “receive” the intervention differently because it has multiple components that can be engaged with in different ways and because users engage with it for as long and as frequently as they choose [47,48] (vs completing a course). It may lead to improvement in relationship quality through complex causal pathways, and its use and effectiveness may be shaped by the social and relationship contexts and settings in which it is used [49]. It is also complex because it requires unaided use of technology [50] (ie, users’ devices and the app), relying on digital literacy skills that may differ between users.

To evaluate *Paired*, we drew on established guidance for evaluating complex interventions [50]. Evaluations can address a range of related questions, such as whether, in what contexts, and for whom an intervention works [51]; how it works; and how it may be further developed. Mixed methods and interdisciplinary approaches are recommended for evaluating complex interventions [52], specifically digital interventions [53-55]. The integration of qualitative and quantitative approaches can aid an understanding of user behavior and issues affecting intervention success [56] and strengthens the conclusions that can be drawn [57]. Qualitative research aids the development of causal explanations by describing the processes that produce an outcome [58].

Relationship quality, our main outcome, is measured in various ways [59]. We believe it is multidimensional [60] (ie, it entails factors such as communication quality and how couples deal with conflict) and is best assessed as such (information received from Di Martino et al [email, January 16, 2025]). In this study, we use a broad definition of relationship quality: “how positive or negative individuals feel about their relationship” [61], which acknowledges that it is subjective yet measurable at the individual level.

We developed a provisional theory of change to guide our evaluation, representing how we expected that *Paired* might “work” based on findings from relationships research and behavioral science. This included the following 3 strands:

- The functionality, design, and content of the app may prompt or facilitate within-couple communication about the relationship (links between improved couple communication and improved relationship quality are well substantiated [62]).
- Daily notifications and in-app interactions may prompt or facilitate daily conversations and “relationship work” by the couple, which may help users learn and advance relationship maintenance skills, including daily gestures [43], benefiting the relationship.
- “Dose-response” (where “dose” is duration and frequency or intensity of use): greater use of *Paired* may lead to greater improvements in relationship quality.

We sought to develop this provisional theory through our research. We consider the role of *Paired* within relationships from a digital sociology perspective in the Discussion section.

#### Aim and Objectives

This study aimed to explore the potential for an mHealth intervention to benefit relationships and how it might do so, using *Paired* as an exemplar. The objectives were (1) to assess the app’s impact on relationship quality by examining associations between relationship quality and duration, frequency and intensity of use, and by comparing the perceived impact of *Paired* among its users (as indicative of the direction of causation); and (2) to develop a refined, empirically informed understanding of how *Paired* may deliver improved relationship quality. To put it simply: (1) “Does it appear to work?” (2) “How does it work?”

## Methods

### Study Design and Participants

The app was evaluated using mixed methods among users of *Paired*. Quantitative and qualitative datasets were analyzed separately, and the findings were integrated (parallel design). Objective 1 was addressed using primarily quantitative approaches, with qualitative data providing explanatory depth and evidencing the likely direction of causation. Objective 2 was addressed primarily qualitatively, supplemented by quantitative findings, because this objective is exploratory. In concordance with our aim (to explore the intervention’s potential), we make no claim of generalizability to all *Paired* users, and our sampling methods reflect this.

### Recruitment, Sampling, and Procedures

Data collection materials, including the wording of survey items, are provided in Multimedia Appendix 1.

#### Brief in-App Survey

At 3 time points with 1-month intervals, starting October 30, 2020, the *Paired* weekly quiz was replaced by a 5-item in-app survey, identified to users as university-led research. This brief survey asked for agreement or disagreement (on a 5-point Likert scale) with 4 statements about aspects of relationship quality (ie, communication, emotional connection, conflict, and sex and intimacy). One further statement concerned participants’ perception of the impact of *Paired* on their relationship communication. Researchers obtained a deidentified extract of data from *Paired* users who had completed at least 1 brief survey. This included data from a “relationship check-up” that users can complete when they first download the app, containing 4 statements similar or identical to those in the brief survey, which we used as baseline measures (the data extract also included demographic details for a small minority of users, which were not used). From 3717 unique participants, the sample was restricted to 440 (11.84%) individuals who completed the relationship check-up in October 2020 and all 3 brief surveys thereafter (4 data points), with at least 7 days between each data point to allow for a realistic prospect of change (in explanation, the data showed that some users delayed completion of the brief surveys; if they completed, say, November’s survey just before completing December’s survey, we would not expect to detect much change). This gave complete longitudinal data spanning approximately 3 months (October-December 2020), referred to as “brief survey data” for brevity.

#### Web-Based Survey

A 30-item survey, administered in December 2020 and hosted securely outside the app, collected quantitative and limited free-text data. *Paired* users were invited to complete it via 3 in-app messages containing the survey link. Those willing to be contacted regarding a research interview provided email addresses.

The survey collected demographic data but not race or ethnicity, religiosity, income, or socioeconomic status (despite well-documented associations with marital satisfaction and quality in the United States [63-65]). These constructs are differently defined and delineated in different cultures, posing challenges for our relatively small-scale international study.

From the web-based survey data, we obtained self-reported duration and frequency of *Paired* use, as well as relationship quality, measured by (1) the Multidimensional Quality of Relationship Scale (MQoRS) and (2) direct questions about relationship quality change since using the app. The MQoRS, developed for this study, combines and weights responses to 17 statements on 5 aspects of relationship quality and is expressed on a scale ranging from 0 to 10. Suited to smartphone-based self-completion (ie, with relatively few survey items comprising simple statements and with responses using 5-point Likert scales that fit on the smartphone’s screen, thus requiring minimal scrolling), it overcomes the shortcomings of unidimensional relationship quality measures and is described in detail elsewhere (Multimedia Appendix 2).

#### In-Depth Interviews

Individual interviews were conducted on the web with purposively sampled web-based survey participants (January-April 2021). The interviewer (TW) identified himself as a university researcher, independent from *Paired*. The primary sampling criteria were gender and country, with initial targets of 5 women and 5 men each from the United States and the United Kingdom. The secondary sampling criteria, by which we sought diversity across the sample, were sexuality, relationship duration, cohabitation, and the presence or absence of children in the household. The interviews followed a topic guide and lasted 40 to 75 minutes. Similar descriptions of *Paired* use and its impacts were heard repeatedly toward the end of data collection, suggesting that saturation had been reached.

### Analyses and Integration

#### Objective 1: Impact of Paired on Relationship Quality

Quantitative data were analyzed using SPSS software (version 26.0; IBM Corp) for descriptive statistics and R software (R Foundation for Statistical Computing) for inferential statistical analyses and data visualization. Statistical significance was considered as *P*<.05.

Brief longitudinal survey data were analyzed using multilevel multinomial logistic regression models with cumulative logit link functions. Predicted values were used to generate odds ratios (ORs) and 95% CIs for change in each dimension of relationship quality over time compared to baseline (the initial “relationship check-up”).

Drawing on data from the web-based survey, MQoRS scores were compared between different categories of reported duration and frequency of using *Paired*, using 1-way ANOVAs with Welch approximation due to unbalanced sample sizes and post hoc Tukey tests. Responses to direct questions about change in relationship quality since using *Paired* were compared using chi-square tests to make binary comparisons between people reporting using *Paired* for ≥1 month versus <1 month and people reporting using *Paired* on 6 to 7 days per week versus ≤5 days per week (among those reporting <1 mo use). Of note, because we lacked the data to predict our sample and subsample sizes in advance, decisions on the cutoffs for these categories were not made a priori but were data driven and were also informed by our provisional theory of change.

Evidence from the 2 surveys on the association between relationship quality and the duration and frequency of *Paired* use was triangulated. The direction of causation of this association was explored qualitatively, informing the development of the theory of change (objective 2).

We compared the perceived effectiveness of *Paired* between demographic groups, using the proportions agreeing with statements in the web-based survey (detailed in the Results section and Multimedia Appendix 3). We used chi-square tests for association and univariable logistic regression to obtain ORs for demographic differences (using the characteristics we used for interview sampling and additionally age, reflecting widespread age-related assumptions regarding technology engagement). Analysis was restricted to the 514 participants who reported ≥1 month’s use of *Paired* (because it is perhaps less plausible that change in relationship quality among new users is attributable to *Paired*). Qualitative evidence to help explain quantitative findings was provided by interview responses to direct questions and spontaneous remarks about the app’s inclusivity.

#### Objective 2: Theory of Change

TW and CA, both experienced qualitative researchers, conducted the qualitative analysis. We used reflexive thematic analysis [66] to describe how *Paired* seems to work, using a largely inductive process but also informed by the provisional theory of change. “Dosage” and its association with relationship quality were explored quantitatively (refer to the Objective 1: Impact of Paired on Relationship Quality subsection) and qualitatively.

### Ethical Considerations

Ethics approval was provided by The Open University Human Research Ethics Committee (brief survey including linked data: HREC/3759/Gabb; web-based survey and interviews: HREC/3797/Gabb). After reading the study information, web-based survey participants indicated their informed consent on the web before survey completion and reconfirmed this afterwards. They were offered the chance to win one £100 e-voucher (or equivalent value, ie, US $135) as a thank you. Individual interview participants provided oral informed consent. The interviewees were sent an e-voucher worth £20 or US $27 as a thank you.

## Results

### Sample Descriptions

Of the 3717 brief survey participants, 440 (11.84%) provided complete data suitable for longitudinal analysis.

A total of 745 participants completed the web-based survey, with diversity by relationship type and duration, sexual orientation, age, relationship status, and parenthood. Comparison with aggregate data from *Paired* suggests that this sample was broadly representative of *Paired* users (Multimedia Appendix 4).

Within the sample of 20 interviews, quotas were filled, and diversity was achieved by all secondary sampling criteria (Table 1). With each quote, we provide age and sampling characteristics.

**Table 1:** Qualitative interview sample (N=20).

Sampling criteria and categories			Interviewees, n (%)
**Primary sampling criteria**			
	UK woman		5 (25)
	UK man		5 (25)
	US woman		5 (25)
	US man		5 (25)
**Secondary sampling criteria**			
	**Sexuality**		
		Heterosexual	14 (70)
		LGBTQ+^a^	6 (30)
	**Relationship duration (y)**		
		≤1	6 (30)
		1-5	8 (40)
		>5	6 (30)
	**Living with partner? (all married or civil partnered couples were cohabiting)**		
		Yes (cohabiting)	14 (70)
		No (living apart together)	6 (30)
	**Children aged <18 y living in household?**		
		Yes	6 (30)
		No	14 (70)

^a^LGBTQ+: lesbian, gay, bisexual, transgender/trans, queer, and other sexuality and gender minoritized individuals.

### Objective 1: Paired Use and Relationship Quality

#### Overview

**Figure 1:**
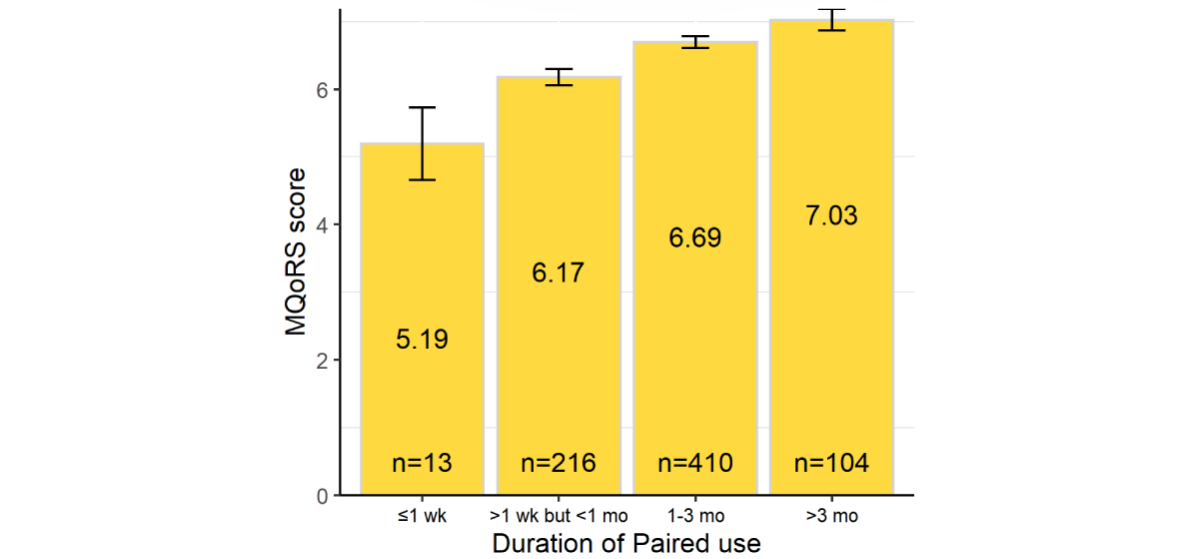
Multidimensional Quality of Relationship Scale (MQoRS) scores by duration of Paired use (showing SDs).

Web-based survey results show that relationship quality, measured by the MQoRS, was 35.5% higher among those with >3 months’ *Paired* use compared to new users (5.19 among people who reported having used the app for ≤1 wk vs 7.03 among those who reported >3 mo use; 95% CI 31.1%-43.7%; *P*=.002; Figure 1). Supporting this, 59.5% (440/740) agreed or strongly agreed that their relationship felt stronger since using *Paired* (95% CI 56%-63%), and people who had been using the app for ≥1 month were more likely to report this than newer users (330/513, 64.3%, 95% CI 60.2%-68.4% vs 110/227, 48.5%, 95% CI 42%-55%; *P*<.001).

**Figure 2:**
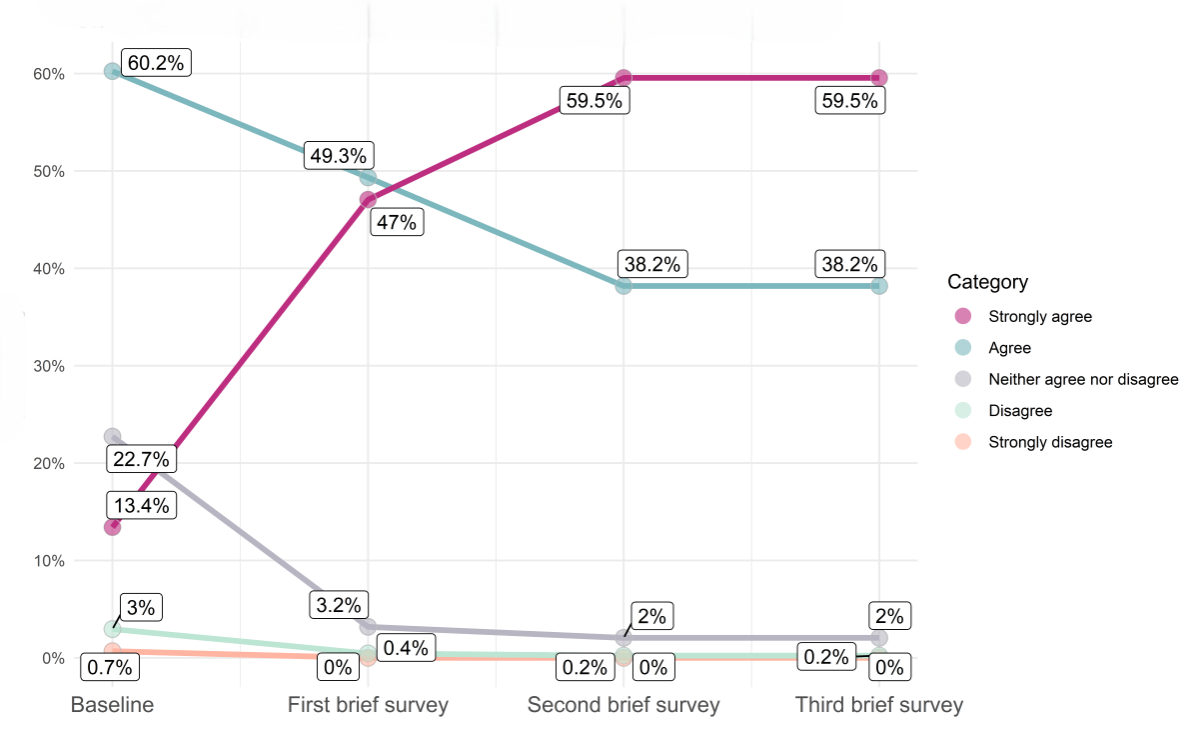
Change over time in responses to the statements “I am very satisfied with how we communicate with each other” and “We communicate openly with each other.”

**Figure 3:**
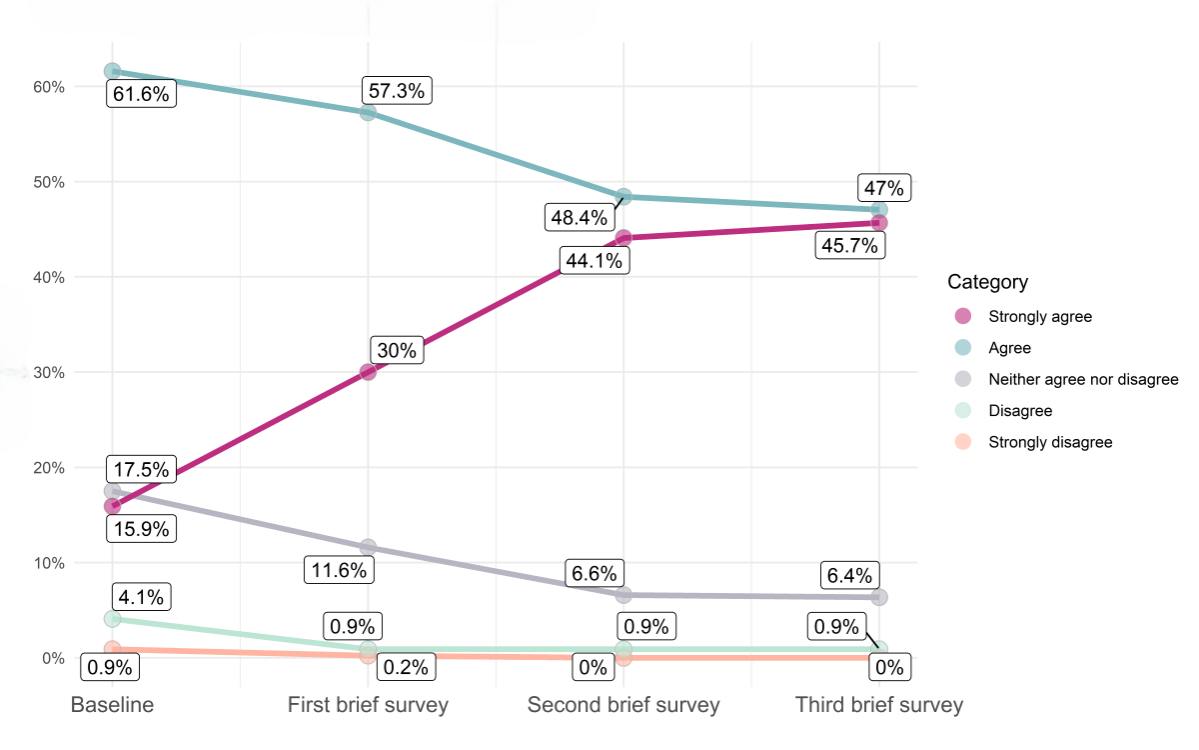
Change over time in response to the statement “We are able to discuss and resolve conflict.”

**Figure 4:**
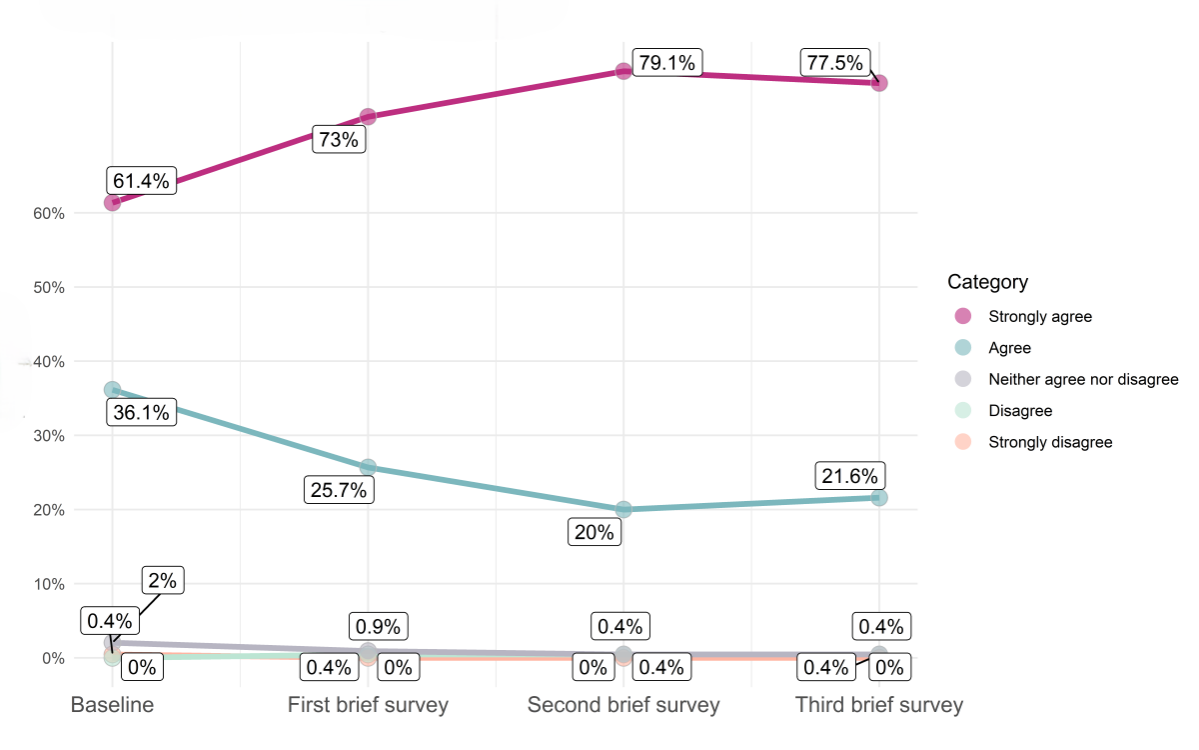
Change over time in responses to the statements “I feel connected with my partner emotionally” and “We enjoy a positive emotional connection.”

**Figure 5:**
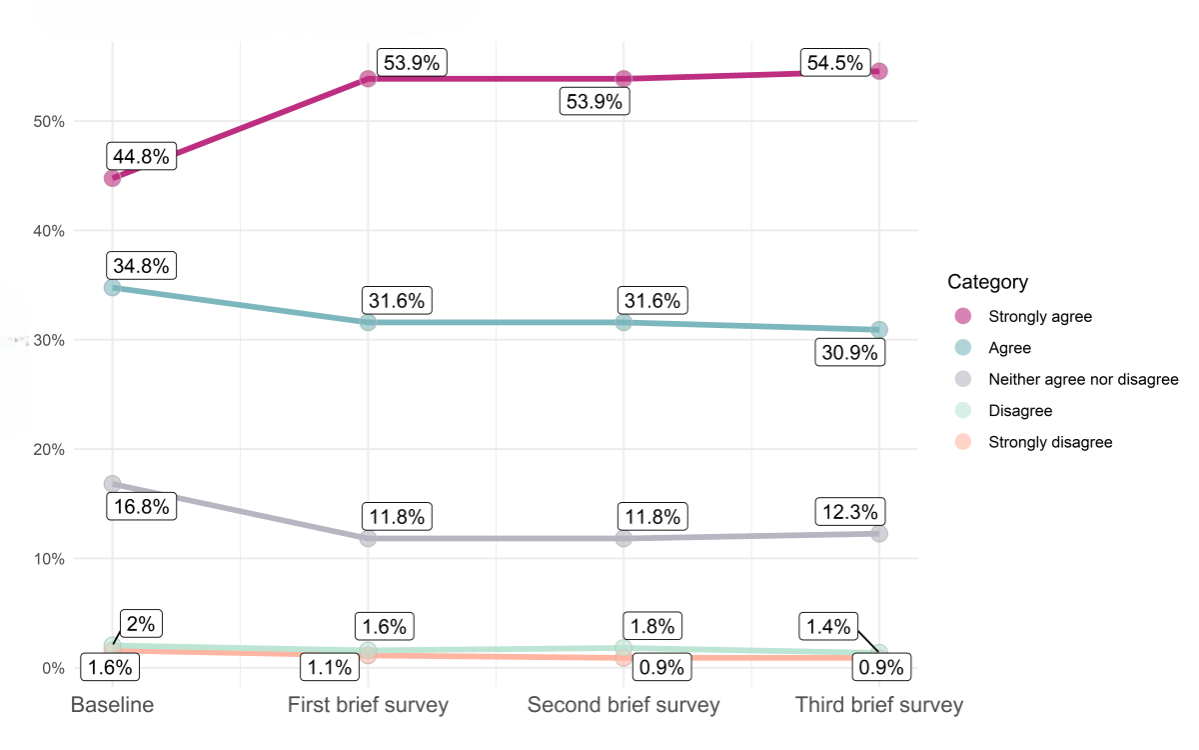
Change over time in response to the statement “We are comfortable with discussing our sex life (with each other).”

The longitudinal brief survey data support this dose-response finding, showing positive changes over time in a cohort of *Paired* users in 4 distinct aspects of relationship quality (Figures 2-5; for each data point in Figures 2-5 [x-axis], the percentages sum to 100%; an increase in the extent of agreement is particularly marked in Figures 2-4, where there is an increase in the percentages responding “strongly agree”; the decreasing percentages responding “agree” represent a shift toward this stronger level of agreement [and not a decline in overall agreement]). The web-based survey analyses also support this (Multimedia Appendix 3).

**Figure 6:**
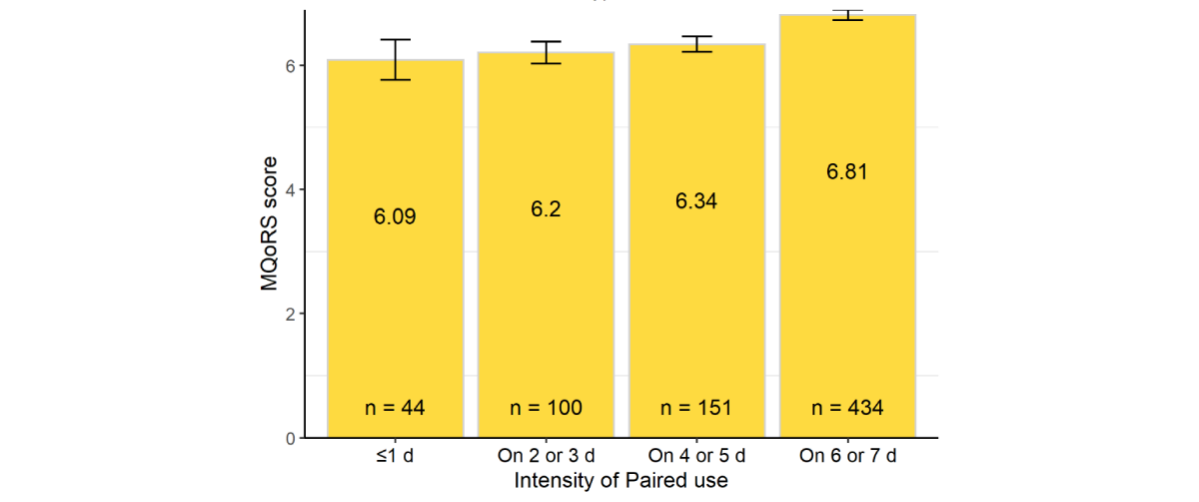
Multidimensional Quality of Relationship Scale (MQoRS) score by number of days using Paired in a typical week (showing SDs).

Reporting more frequent *Paired* use was associated with higher MQoRS scores: people using *Paired* on 6 to 7 days per week had 11.8% higher relationship quality than those using *Paired* on ≤1 day per week (MQoRS scores: 6.81 vs 6.09), and this difference was statistically significant (95% CI 0.19-1.42; *P*=.04; Figure 6; details in Multimedia Appendix 2). Supporting this finding, among people who had used *Paired* for at least 1 month, those who reported using it on 6 to 7 days in a typical week were more likely to agree or strongly agree that their relationship felt stronger since using *Paired* compared to those who used it less often (223/316, 70.6%, 95% CI 65.5%-75.6% vs 106/196, 54.1%, 95% CI 47.1%-61.1%; *P*<.001). However, we found no statistically significant association between the total amount of time spent using *Paired* in a typical week (intensity of use) and MQoRS scores.

#### Comparison of Perceived Effectiveness by Demographic Characteristics

Among those who reported having used Paired for ≥1 month, there was no statistically significant difference in the proportion of web-based survey participants who agreed or strongly agreed that “Paired is improving how we communicate as a couple” (overall 414/514, 80.5%), “Our relationship feels stronger since we’ve been using Paired” (overall 330/513, 64.3%), or “The longer I use Paired, the better my relationship gets” (overall 252/514, 49%) between men and women or by age group, sexuality, country, relationship status, or relationship duration (refer to Multimedia Appendix 3 for details of the demographic categories used). Participants without children (aged <18 y) in their household were more likely to agree that their relationship felt stronger since using *Paired* compared to those living with children (OR 1.49, 95% CI 1.00-2.21), but this was of borderline statistical significance (*P*=.048), and no differences were observed in agreement with the other statements.

Regarding the qualitative data, the inclusivity of *Paired* was described positively; for example, it was described as suitable for a range of ages (“kind of age agnostic” [UK02, woman, aged 58 y, heterosexual, relationship duration: ≤1 y, living apart, no children]) and as potentially benefiting couples in different ways over the course of their relationship (“as a prompt of finding out more about your partner early on, or [for] mature couples, the stuff you take for granted” [UK07, man, aged 36 y, heterosexual, relationship duration: >5 y, cohabiting, children]). While some commented that some of the app’s content did not apply to their relationship (eg, sharing domestic chores among noncohabiting couples), this was not necessarily perceived negatively. Interviewees valued questions and content that were broadly relevant to diverse couples. As an interviewee in a same-sex relationship explained, he had never come across a question which was not inclusive:

I’ve never furrowed my brow at a question, [and never thought] oh my god how heteronormative, how cis, clearly it’s a bunch of cisgendered white men programming this app.

US01, man, aged 34 y, LGBTQ+ (lesbian, gay, bisexual, transgender/trans, queer, and other sexuality and gender minoritized individuals), relationship duration: ≤1 y, cohabiting, no children

We’ve found it more useful than faith-based apps. And I think sometimes you can be a Christian couple that has conversations. You don’t have to be a Christian couple that has Christian couple conversations.

UK10, man, aged 27 y, LGBTQ+, relationship duration: 1-5 y, living apart, no children

Another interviewee considered *Paired* “just so useful for anyone” but wondered whether some people would be reticent to start using it:

...there’s plenty of those “typical guys” who are going to be like, “I don’t need no stupid app to get to know this and this” [...] But I feel like once someone gets into it and starts using it and understands how beneficial it is that it can pretty much benefit anyone.

US04, man, aged 38 y, heterosexual, relationship duration: >5 y, cohabiting, no children

### Objective 2: Refined Theory of Change

#### Overview

A total of 4 themes were identified from the qualitative analysis, 3 (75%) of which drew upon the provisional theory of change (refer to the Approach and Theoretical Basis subsection in the Introduction section). Subthemes and the theme *Engagement* (which describes how *Paired* use is sustained) were derived from the data. Theme and subtheme descriptions (subheadings) in this subsection summarize the refined theory of change.

#### Communication: Practicing Communication to Strengthen Emotional Connection and Improve Relationship Communication Skills

##### The App Is a Neutral Prompt for Relationship Conversations

Interviewees found it uncomfortable to raise difficult topics and felt as though they were nagging their partner by doing so. The following interviewee describes the consequences of this:

...a much more tense, stressful situation to talk about, than “this wasn’t on my mind but the app brought it up so why don’t we talk about it?”

US06, woman, aged 44 y, LGBTQ+, relationship duration: 1-5 y, cohabiting, children

Without the app, couples might avoid discussing a challenging topic until circumstances meant that it had to be addressed, whereupon they might struggle to communicate without arguing:

[Discussions about bills] used to be sort of heated arguments. [But] Paired allowed us to kind of discuss such things more easily and freely because we were discussing them in the context of a question that it says, discuss.

UK05, man, aged 45 y, heterosexual, relationship duration: >5 y, cohabiting, children

By interspersing serious and lighter questions, relationship communication became easier. As an interviewee described, this brought “balance” to relationship communication between her and her partner:

A lot of the questions that the app has are ones we can talk about easily without one of us getting upset, and it makes it easier to talk when you don’t dread it.

US07, woman, aged 23 y, heterosexual, relationship duration: 1-5 y, cohabiting, no children

##### Questions and Quizzes Help Couples Get to Know Each Other Better, Stay Connected, and Refresh or Deepen Intimacy

Interviewees described how the app’s daily questions and quizzes helped them to gain new insights about their partner and their relationship, which informed changes to their own behavior:

...it’s definitely given me more insight on his position, how he feels [...] I didn’t realize he was really not sure about how he expresses his feelings, so now I can take some time and try to really draw that out before we discuss.

US07, woman, aged 23 y, heterosexual, relationship duration: 1-5 y, cohabiting, no children

Question and quiz responses could remind couples of what they shared:

It’s funny there was one question about a favorite memory of the year [...] [We wrote] the exact same memory. Yeah, I think it has been reassuring. It has reinforced our already strong connection.

US06, woman, aged 44 y, LGBTQ+, relationship duration: 1-5 y, cohabiting, children

This enabled interviewees to gain “new insights” about themselves and each other and to “expand and communicate better” through questions coming from the app, a “third party” (UK04, woman, aged 23 y, LGBTQ+, relationship duration: >5 y, living apart, no children). It helped new couples get to know each other, and long-term couples to become “better connected.” For example, an interviewee noticed his wife’s responses to some of the app’s questions changing over time, which, he felt, helped sustain the connection between them:

[I’m] no longer keeping this stale, unevolving picture of who she is and I think that is the key to being connected to someone, to remain connected.

US04, man, aged 38 y, heterosexual, relationship duration: >5 y, cohabiting, no children

As a way of maintaining emotional connection, *Paired* was welcomed by couples in contrasting contexts. This included physical separation (eg, a long-distance relationship, COVID-19 quarantine, or self-isolation) and being in lockdown together. An interviewee described how while he and his wife were working from home and homeschooling their children together, his wife was “just overstimulated with communication that she already has with the kids and work and stuff like that.” He explained how using *Paired* helped improve the frequency and quality of their relationship communication, making it “easy to just keep that tank topped up a bit” when they were “very much in survival mode” (UK07, man, aged 36 y, heterosexual, relationship duration: >5 y, cohabiting, children).

##### Relationship Communication Skills Are Learnt and Developed Through Regular Practice, Sometimes Supplemented by Guidance

By regularly responding to and discussing questions and quizzes, accessing content and tips, and putting their learning into practice, couples developed their relationship skills. They observed how these skills improved. One interviewee stated that “the communication is so much better and it’s stopped a lot of shouting at each other,” and she went on to explain how when arguments begin, they are more able to stop and give each other space before apologizing; app notifications could distract the couple from an argument, prompting them to engage with *Paired*, which “just changes the whole atmosphere again, which is just amazing” (UK09, woman, aged 32 y, heterosexual, relationship duration: 1-5 y, cohabiting, children).

Within and between couples, there was a wide range of use of the additional content and resources that *Paired* provides: some read widely, others rarely accessed these parts of the app (instead mostly using questions and quizzes). Some shared what they found with their partner:

If maybe we’re like not super aligned on [daily questions], I know sometimes they give you tips so I’ll read it and I’ll be like hey read this [...] having that knowledge in the background is more helpful if it comes to a conflict later.

US10, woman, aged 25 y, heterosexual, relationship duration: ≤1 y, living apart, no children

#### Engagement: Getting Hooked on the App, Getting Hooked on Each Other

##### Being Interested and Curious About New Content—and About Each Other

Regular engagement is prompted via the app (eg, automated in-app messages and daily questions) and by partners reminding each other to complete questions or quizzes or sharing articles. Interest and curiosity provoked their engagement:

I’ll get notifications that she has answered it and I want to see what she’s said so I go onto my one.

UK06, man, aged 33 y, LGBTQ+, relationship duration: 1-5 y, cohabiting, no children

Because it is a pleasant interaction [...] I’m curious what today’s question is. You know, if my partner’s gone into it before me [...] it’s like, ooh what did they answer? Or, I wonder what they will answer, let me put mine and that will nudge them to get their answer in.

UK07, man, aged 36 y, heterosexual, relationship duration: >5 y, cohabiting, children

##### Fun and Entertainment

Lighthearted questions helped make *Paired* enjoyable and facilitated engagement in relationship work:

I think [the questions are] fun and I think they’re informative and I think the way they’re done is pretty low pressure. Like I’m never scared, like “oh I have to put the right answer, it’s scary.” I think they’re pretty low effort but still can lead to good conversations.

US10, woman, aged 25 y, heterosexual, relationship duration: ≤1 y, living apart, no children

As one interviewee explained, she and her partner enjoyed the quizzes the most:

...the questions sometimes are very informative. But when we have the quizzes it feels really easy to compare our views on things. So I asked my partner yesterday how do you actually feel about this app [...] he definitely said “oh quizzes, yeah I love quizzes, it’s just so fun.”

UK01, woman, aged 18 y, heterosexual, relationship duration: ≤1 y, cohabiting, no children

Another interviewee mentioned a Christmas-themed quiz, accessed at a time of year which was often “stressful” for the couple:

...it was nice to have something to talk about that wasn’t stressful, and that was little. Like “what is a holiday tradition that you want to do with your kids?” [...] It’s not tiny because it’s important, but it’s also not like “how are we going to get [partner’s parent] to respect me with our child?” [...] it’s a little more chill.

US07, woman, aged 23 y, heterosexual, relationship duration: 1-5 y, living apart, no children

While some interviewees sought out quizzes within *Paired* to complete with their partner for fun, others found the “fun” questions and quizzes too trivial:

Sometimes it’s not serious enough but maybe we’re just too serious.

UK10, man, aged 27 y, LGBTQ+, relationship duration: 1-5 y, living apart, no children

As such, there was a tension between this subtheme and the following subtheme.

##### Experiencing Meaningful Benefits and Wanting More

Experiencing short- and longer-term benefits was a key motivator for sustained *Paired* use. These benefits ranged from enjoying a moment of connection to looking back over past months and recognizing that relationship communication had improved (refer to the theme *Communication*). This created a virtuous cycle, where benefits attributed to the app led couples to continue using it.

##### Maintaining a “Streak” Can Be a Motivator and Signifier of Commitment

*Paired* informs users of their “streak” (number of continuous days of answering daily questions). For some, the streak was unimportant, while for others it was a motivator to engage daily:

I’ve almost never found myself not in the mood to answer one of these questions [...] because I get a lot out of it, conversely there’s time where my wife destroys her streak only because of the fact that she’s just like, either she was too busy or whatever, “I just couldn’t be bothered doing it today.” Where I’m just like, this is fun!

UK07, man, aged 36 y, heterosexual, relationship duration: >5 y, cohabiting, children

Partners can see how long each other’s streak is, which motivated some but was off-putting for others: a partner’s lower streak could be perceived as signifying lower commitment to the relationship:

God forbid I miss my streak and have to start over, that’s my personality. But when I look at the app and I see that I’m on a streak of 27 days and he’s on 2, it gets to me [...] feeling like he’s not as committed to working on our relationship. I think maybe [the streak’s] good for some people and it’s reassuring and it’s motivating, but for us it just causes more problems.

US06, woman, aged 44 y, LGBTQ+, relationship duration: 1-5 y, cohabiting, children

#### Dailiness: Paired Use and Regular Relationship Communication Can Become Embedded in Couples’ Daily Lives

##### App Use Can Become Habitual

Through regular engagement, *Paired* use could become a pleasant habit, which was therefore sustained:

...it’s just become a habit now [...] automatically just do it at a certain time of the day. Knowing [...] this is what we’ll be discussing shortly. I think we both kind of look forward to having stuff to talk about that is not work, not kids, not finances.

UK05, man, aged 45 y, heterosexual, relationship duration: >5 y, cohabiting, children

##### Regular Relationship Communication Can Become Habitual

Regular relationship communication prompted by the app can become part of couples’ intimate lives because it “prompts conversations [...] forces you to talk about something proper every day” (UK01, woman, aged 18 y, heterosexual, relationship duration: ≤1 y, living apart, no children). Interviewees described how they tended to respond to the questions and discuss them at particular times in their daily routines:

I usually come out into the living room because I wake up earlier most days [...] And when he answers his Paired question is how I know he’s awake.

US01, man, aged 34 y, LGBTQ+, relationship duration: ≤1 y, cohabiting, no children

This became regular time devoted to the relationship:

Initially we would talk about Paired in the morning but probably I’m already in work mode. Or we’d talk about Paired at lunchtime and eventually we realized it was probably best to talk about it just before bed. When we were together, relaxed, just before we start bingeing on our box sets.

UK05, man, aged 45 y, heterosexual, relationship duration: >5 y, cohabiting, children

Discussions occurred in person, by telephone, SMS text message, or on the web (eg, Snapchat, Instagram, or WhatsApp). Users came to expect and look forward to daily relationship communication:

[Paired] creates these little windows and pockets of time for us to talk about something that has a benefit or is something silly or makes us laugh or something that just reinforces that it’s difficult, but it’s not really that difficult.

UK08, man, aged 44 y, heterosexual, relationship duration: >5 y, cohabiting, children

#### “Dosage”: Regular Use of Paired Delivers Incremental Benefits

The theme *Communication* described how *Paired* can cumulatively improve relationship quality through prompting meaningful conversations and providing topics, enabling couples to regularly practice communication and enhance their emotional connection. In the themes *Engagement* and *Dailiness*, we described how the use of *Paired* can be sustained and become embedded in couples’ daily lives. Supporting the quantitative findings, interviewees noticed gradual, incremental gains with regular, frequent use of the app:

...I’m struggling to find something that’s major and has left an imprint. But the bottom line is all those interactions have had a positive impact.

UK07, man, aged 36 y, heterosexual, relationship duration: >5 y, cohabiting, children

The survey findings showing that the amount of time (per week) spent on the app was relatively unimportant may be explained by the qualitative findings that *Paired* facilitates relationship maintenance behaviors that may occur offline:

...it’s a little time spent [on the app] for a lot of love gained.

UK10, man, aged 27 y, LGBTQ+, relationship duration: 1-5 y, living apart, no children

## Discussion

### Principal Findings

The regular use of an mHealth intervention, specifically *Paired*, seems to improve relationship quality over a relatively short time frame. The MQoRS score was 35.5% higher among people reporting >3 months’ use of *Paired* compared to new users (≤1 wk), with longitudinal data showing similar positive trends. Regular, daily use benefited relationships the most and did not require intensive use of the app. The interview data suggest that this may be due to how the app prompts enjoyable and meaningful conversations, and these interactions help couples develop and practice relationship maintenance skills and feel more emotionally connected. Using *Paired*, and the habits it engenders, can become embedded in couples’ daily lives as they look forward to their partner’s response to the daily questions and make time for regular relationship communication. Noticing the app’s impact helps to sustain its regular use in a positive feedback loop (virtuous cycle).

### Strengths and Limitations

Our study was relatively small scale (eg, the web-based survey sample included just 13 new users of the app [≤1 wk use], which is something we could not have anticipated). Data collection occurred over 3 months when there were no major changes to the app and among people who had chosen to use it. This self-selected sample may be more open to positive relationship care and more digitally literate than the general population, which may limit the transferability of our findings; however, our international sample is a strength. Regular users may be overrepresented among participants because they were more likely to see the research invitations and more likely to provide complete longitudinal brief survey data. These factors and our sampling methods preclude generalization to all *Paired* users but are unproblematic to the study’s aim of exploring the *potential* of a stand-alone mHealth intervention in relationship care.

Our study design is appropriate for this early stage in the app’s evaluation. As well as providing proof of concept, we developed a theory about how *Paired* works in context. This work could inform a future randomized controlled trial to quantify the app’s effectiveness compared to an alternative (or no) intervention [50]. Future evaluative work will need to take into account the app’s widespread availability and assess its effectiveness during its iterative, ongoing development [53]. Proceeding straight to a trial would have been unnecessary (and potentially wasteful) without the indicative evidence of potential effectiveness, or proof of concept [52,67], that we have now provided.

Our use of mixed methods design is appropriate to our aim of developing an in-depth, contextualized understanding of whether and how a complex digital intervention can “work” in practice. The integration of data from complementary sources increases the validity of the main finding that *Paired* could improve relationship quality. In explanation, analysis of the cross-sectional web-based survey demonstrates that relationship quality (measured by the MQoRS and by responses to direct questions about the app’s impact) is positively associated with the reported duration of *Paired* use. This finding could represent a positive association between relationship quality and duration of use or higher disengagement from the app among people in poor-quality relationships. The latter explanation can be discounted as unlikely because longitudinal (brief survey) data show improvements in various aspects of relationship quality in a cohort of users over 3 months. The interviewees’ descriptions of how *Paired* helped them demonstrate a perceived causal association: use of the app contributes to improved relationship quality. It is possible that analyses based on subgroups of the web-based survey data may be underpowered, although the *P* values (and CIs) obtained suggest that this is unlikely. The broad agreement between the findings obtained from multiple data sources in this mixed methods study provides further reassurance.

We used a multidimensional measure of relationship quality, the MQoRS, which was developed based on theory and evidence derived from the *Enduring Love?* study [43,68,69] using robust statistical analyses to assess its validity and reliability. It overcomes the shortcomings of existing unidimensional scales (acknowledging that relationship quality is a complex construct) and is suited to mHealth research (information received from Di Martino et al [email, January 16, 2025]). The in-app survey was brief to increase the likelihood of repeat completions and thus obtain longitudinal data, which precluded use of the 17-item MQoRS; yet, encouragingly, the results support the same main finding.

The surveys used convenience sampling; the brief survey lacked demographic data; and, during our study, the app’s data on its users’ demographics were very incomplete. Therefore, we could not check the survey samples’ representativeness to users overall, but we made such comparisons as were possible. These were favorable: the web-based survey participants seemed broadly representative of the active users of *Paired* (Multimedia Appendix 4). Although our sample is international, we could not explore use or effectiveness by race, ethnicity, religion, or socioeconomic status because we did not collect these data, for reasons explained in the Methods section. Both survey datasets may include nonindependent data if both partners in a couple participated; however, we cannot account for this, which is a limitation (matching data within anonymous surveys poses feasibility, acceptability, ethical, and privacy issues because asking for participants’ own and their partners’ names renders the data identifiable). The web-based survey data on the duration and frequency of engagement with *Paired* are self-reported and therefore could be subject to recall bias. App-collected metrics would be preferable but were not obtained (this would have raised ethical and data governance issues). However, the longitudinal brief survey data effectively provide the approximate duration of *Paired* use because these surveys were repeated over time in a cohort of app users. Triangulation and integration of data from multiple sources strengthens the conclusions we are able to draw regarding the “dosage” of use of the app.

### Meaning and Implications

#### Digital Relationship Care

The findings provide proof of concept of mHealth for relationship care for supporting modest improvements in relationship quality, over a relatively short time frame, in a self-selected population of users of 1 app. In the context of limited research on fully digital couple interventions for relationship care [45] (ie, positive, preventive interventions that are not blended or therapist guided), our rigorous evaluation makes an important contribution to the evidence base. The findings fit with existing evidence and theory about the importance of regular daily “relationship work” in sustaining couple relationships [43] and demonstrate how this can be supported in practice. The interview accounts of how benefits can accrue from regular, positive interactions and moments of connection evoke the concept of “relationship banking” [70] and demonstrate how mHealth can prompt these interactions. We would not expect large changes in relationship quality, given the many contextual influences on relationships, the time frame, and the fact that the study population included users whose relationship quality was already good (among whom scope for improvement is limited).

#### Inclusivity and Accessibility

Similar perceptions of the app’s effectiveness among people of different ages, sexualities, and relationship types and durations suggest that it is inclusive by these characteristics. Therefore, its appeal is likely to be broad, although ethnic and cultural inclusivity has not been explored. Evidence of the effectiveness of *Paired* is promising in the context of underuse of existing preventive interventions. The convenience and flexibility of mHealth suits the ebbs and flows of couple relationships because partners have control over when and how much they engage and apparently benefit without needing to use the app intensively. Barriers to uptake are low for digitally literate web-based populations, and digital interventions are scalable (eg, the work of creating new questions and content for *Paired* is the same, irrespective of the number of users), contrasting with face-to-face or “blended” courses or therapy, which require conjoint commitment and a professional’s input with each couple or group.

#### Theory and Practice

Where mHealth interventions incorporate tailoring to individual users’ characteristics, this can increase the personal relevance of content and messaging, which in turn can increase engagement with the intervention and improve learning [71]. Blended interventions may achieve similar effects through personalized messaging from a coach to each individual user. We have shown that an intervention without these features can be personally relevant because partners effectively create content for each other. They “receive” an intervention that has a unique human touch (their partner’s) that is not only personalized but intimately personal. Peer-to-peer digital communication ordinarily requires resource-intensive moderation, but this does not apply to *Paired* because communication is between partners only.

Theories of behavior change tend to be individualistic, although some acknowledge the social dimensions of learning [31,72]. The refined theory of change that we developed is novel in encompassing the dyadic nature of behavior change within relationships, and in emphasizing how human-technology interactions and between-partner interactions support this change and sustain engagement. *Paired* engagement is sustained directly (eg, in-app messages) and indirectly (partners remind each other to answer questions; they experience relationship improvements and are motivated to continue to engage).

A combination of fun and meaningfulness seemed to help maintain engagement, and the app prompted regular daily “relationship work.” These findings may inform self-help and professionally delivered courses and therapy, both digital and face-to-face, including future iterations of *Paired*.

#### Digital Intimacy

The use of apps to score and rate users’ sexual lives has been described by Lupton [73] as reinforcing reductive and normative perspectives on what is “good” within an area of intimate life. Levels of agreement in *Paired* quizzes are undoubtedly reductive assessments of how “good” a relationship is, but in contrast to the apps reviewed by Lupton [73], these comparisons are not designed for ranking or sharing outside of the couple. Instead, they stimulate within-couple discussion and interpretation. Daily questions elicit free-text responses, eluding quantification, but “streaks” are comparable between partners. The work conducted by Lupton [55,74] explains how mHealth apps become more-than-human individual-app assemblages, with their own agency. We suggest, based on our interviews, that *Paired* creates an individual-partner-app assemblage, functioning within and as part of couples’ intimate lives [75]; for example, daily question completion indicates to one partner that the other is thinking of the relationship, both documenting and becoming part of couples’ relationship work; differences in streak length may signify lower commitment to the relationship. App-based indicators of conjoint accountability may cause difficulties for couples where problematic relationship dynamics already exist [42]. In a study of young people’s communication regarding their Snapchat streaks, Hristova et al [76] found that not maintaining a streak could have significance for peer relationships similar to what we found for couples (unlike *Paired* streaks, which are individualized, a Snapchat streak is a continuous period of at least daily “snaps” exchanged between a pair of users).

#### Transferability and Relevance

We evaluated 1 app, within the first 3 months of its availability, as an exemplar to explore whether mHealth can benefit relationships. Choosing *Paired* was fortuitous, given the rapid turnover of apps, most of which fail (an estimated 99.5% [77]). *Paired* became the leading relationships app worldwide in terms of revenue and downloads in 2021 and 2022 [78]. It expanded from 10,000 active users during our study to >1 million monthly active users in November 2024 (information received from *Paired* [personal communication, December 10, 2024]). Therefore, the findings inform an app with considerable reach. *Paired* became more sophisticated since data collection, but our findings remain relevant because core features remain, including the daily questions that were so important. The app’s impact may be affected by how new technologies’ user populations tend to be differently constituted over time [79]. Our findings should not be generalized uncritically to apps with different functionality, features, and content. We make no claim that our findings are generalizable to all couples or all users of *Paired*. We have not explored what happens to relationship quality beyond 3 months’ use of *Paired* or whether changes are sustained if couples stop using it.

#### Pandemic Context

This research took place during the COVID-19 pandemic, and couples may have turned to *Paired* during relationship strain; however, the data are not suggestive of severe relationship problems among participants. Media predictions of large increases in relationship breakdown and divorce during the pandemic did not occur [15,80]. Instead, impacts on relationships were heterogeneous—and positive for some couples [17,81]—although long-term impacts remain unknown [82,83]. However, couples’ access to formal and informal relationship support was reduced or interrupted. Some people invested in personal growth and relationships during lockdowns and quarantines [84], often supported by digital resources. Couples may use *Paired* differently now, perhaps preferring to engage with relationship care through other means or not at all. Nevertheless, if the digital engagement with services, social lives, and entertainment that accelerated during COVID-19 is here to stay, interest in digital relationships care may persist—as *Paired* subscription patterns suggest.

### Future Directions

*Paired* may complement existing relationship self-help and support interventions, for example, for couples who are unready or waiting for therapy, as blended care alongside conventionally delivered relationship therapy, or during “offboarding” (after the cessation of therapy). Future work could explore reasons for disengagement with *Paired*, which could be interpreted positively (it has done its job), negatively (it is not working), or neutrally (it would be surprising if any intervention suited everyone). Digital solutions are not a panacea due to inequalities in technology access and digital skills [85]. Further evaluative research could take a theory-based and systems perspective [67] regarding how digital interventions fit within the relationship care and support ecosystem and extending our findings to delineate optimal contexts of use, that is, for which couples, in which circumstances, and how *Paired* may best enhance couple relationships. Data from couple dyads and longitudinal qualitative data would enable an exploration of the influences, effects, and contexts of different patterns of use (eg, daily, weekly, or episodic). The meaning of *Paired* use within relationships has been explored in greater depth in a separate paper [75].

### Conclusions

*Paired* has the potential to improve relationship quality over a relatively short time frame. It does this by being an engaging mHealth intervention that prompts regular daily relationship communication, supporting the development of relationship skills and increasing feelings of emotional connection.

## Appendix

Multimedia Appendix 1 Data collection materials.

Multimedia Appendix 2 Multidimensional Quality of Relationship Scale development, content, and analyses.

Multimedia Appendix 3 Additional statistical analyses.

Multimedia Appendix 4 Comparison of web-based survey participants and Paired users.

## Abbreviations

LGBTQ+: lesbian, gay, bisexual, transgender/trans, queer, and other sexuality and gender minoritized individuals
mHealth: mobile health
MQoRS: Multidimensional Quality of Relationship Scale
OR: odds ratio
