# Supplementary material for: Exploring the Potential of a Digital Intervention to Enhance Couple Relationships (the Paired App): Mixed Methods Evaluation
Source: JMIR Mhealth Uhealth. 2025 Apr 14;13:e55433. doi: 10.2196/55433 (PMC12001865; doi:10.2196/55433)
Supplement: Multimedia Appendix 1 [file mhealth_v13i1e55433_app1.docx]

## Multimedia appendix 1: Data collection materials

### Brief in-app survey and ‘relationship check-up’ quiz wording

In both the initial ‘relationship check-up’ and the brief survey, participants were asked the extent to which they agreed with the statements, on a 5-point Likert scale (strongly disagree, disagree, neither agree nor disagree, agree, strongly agree), which is suited to the smartphone interface.

A fifth statement in the brief in-app survey (but not the ‘relationship checkup’) was: ‘Paired is improving how we communicate as a couple’. It was not used in our analyses as we considered it overlapped with the first statement on communication

| **Area of relationship quality** | **Initial ‘relationship check-up’ statement** | **Brief survey statement** (identical for all three surveys) |
| --- | --- | --- |
| **Communication** | I am very satisfied with how we communicate with each other | We communicate openly with each other |
| **Dealing with conflict** | We are able to discuss and resolve conflict  *(identical wording)* | |
| **Emotional connection** | I feel connected with my partner emotionally | We enjoy a positive emotional connection |
| **Sex and intimacy** | We are comfortable discussing our sex life | We are comfortable discussing our sex life with each other |

### Web-based survey
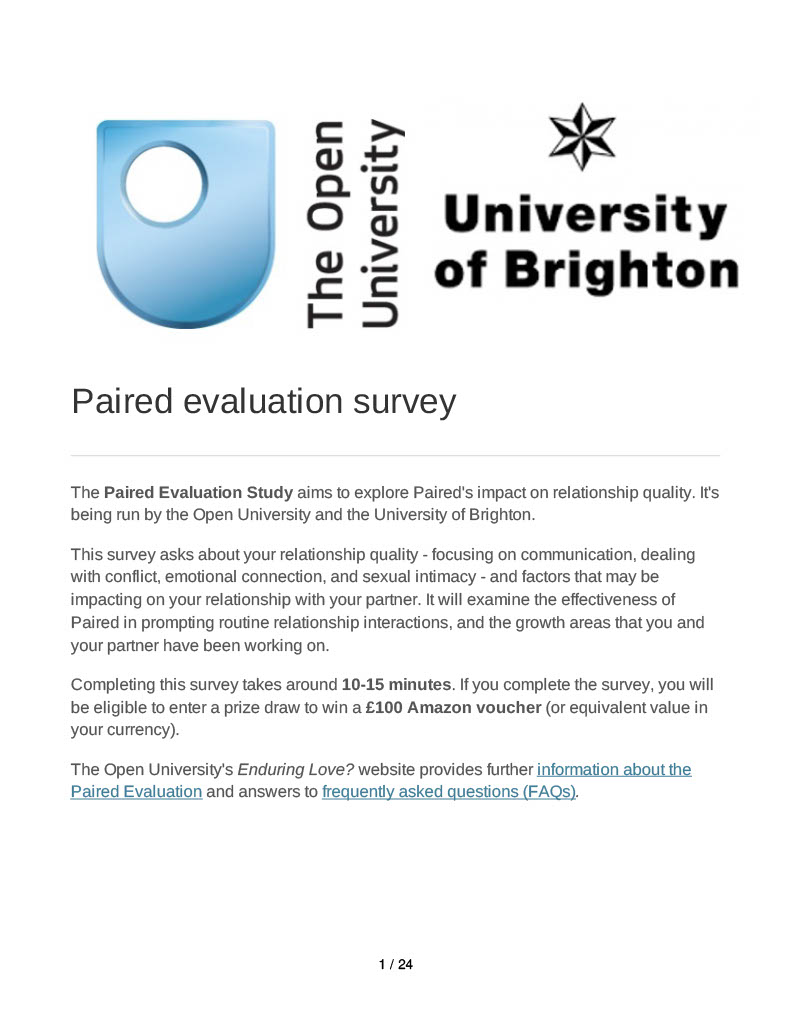

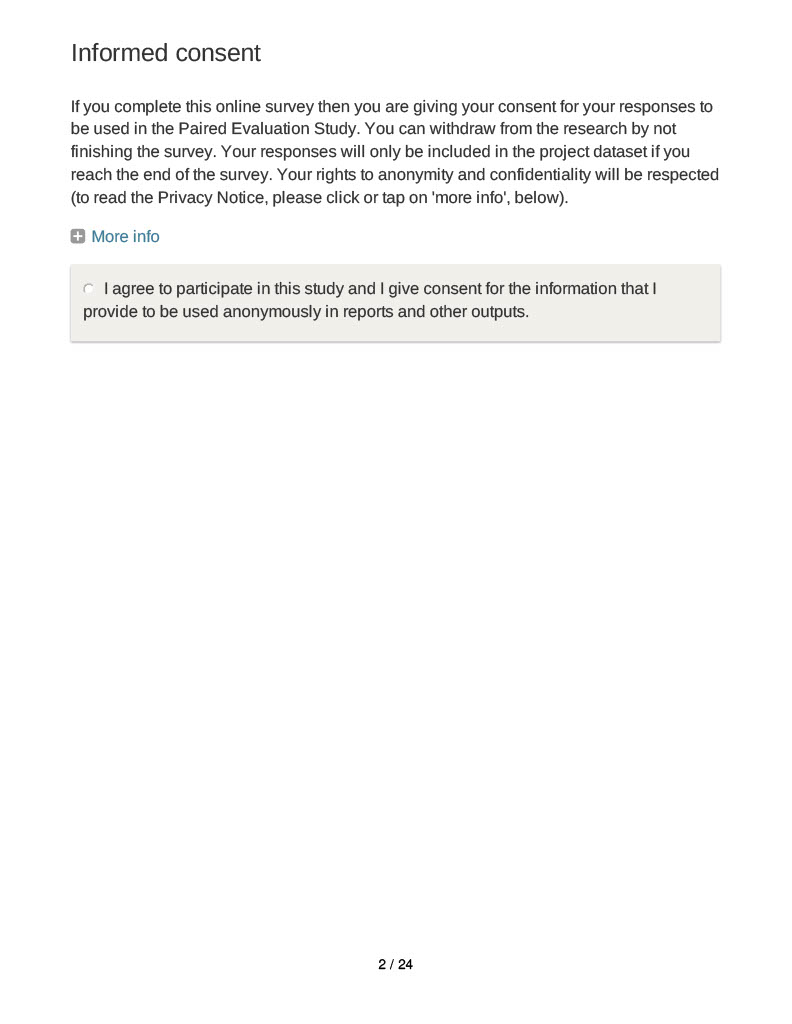

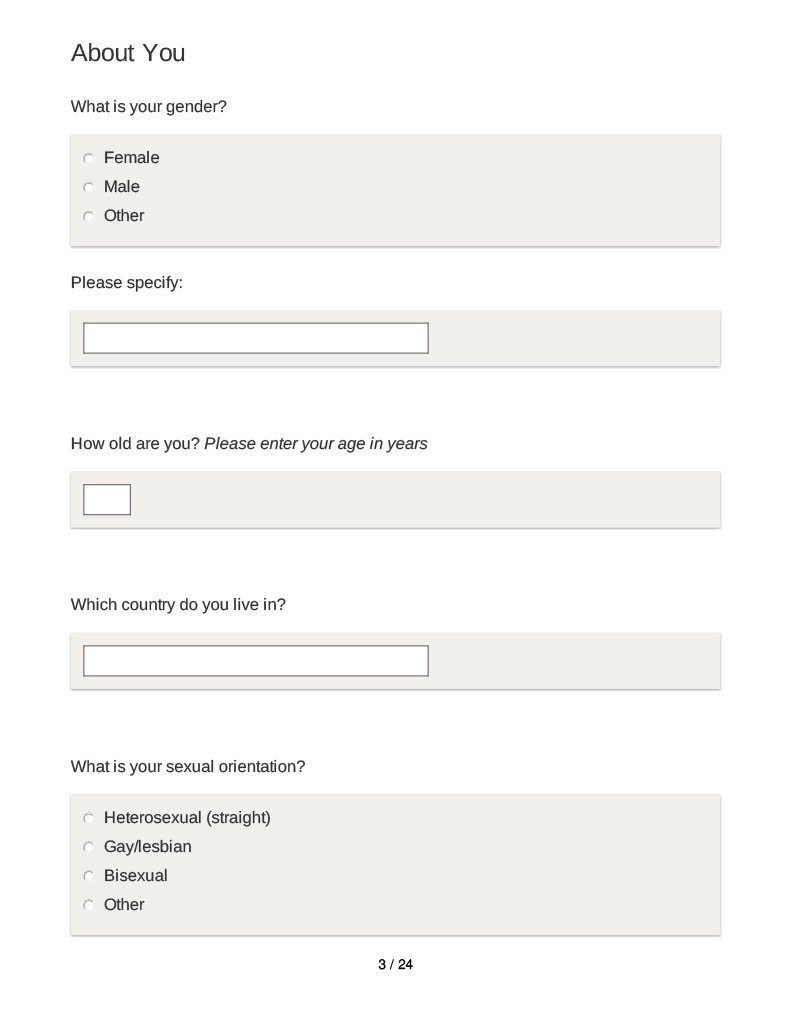

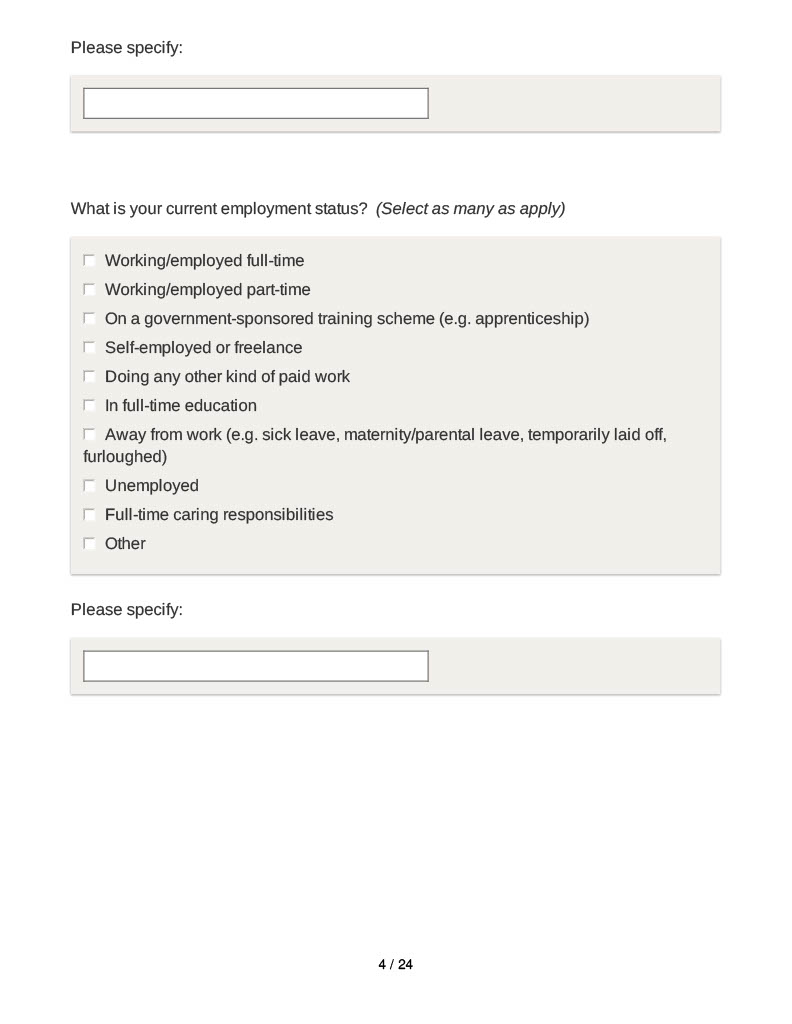

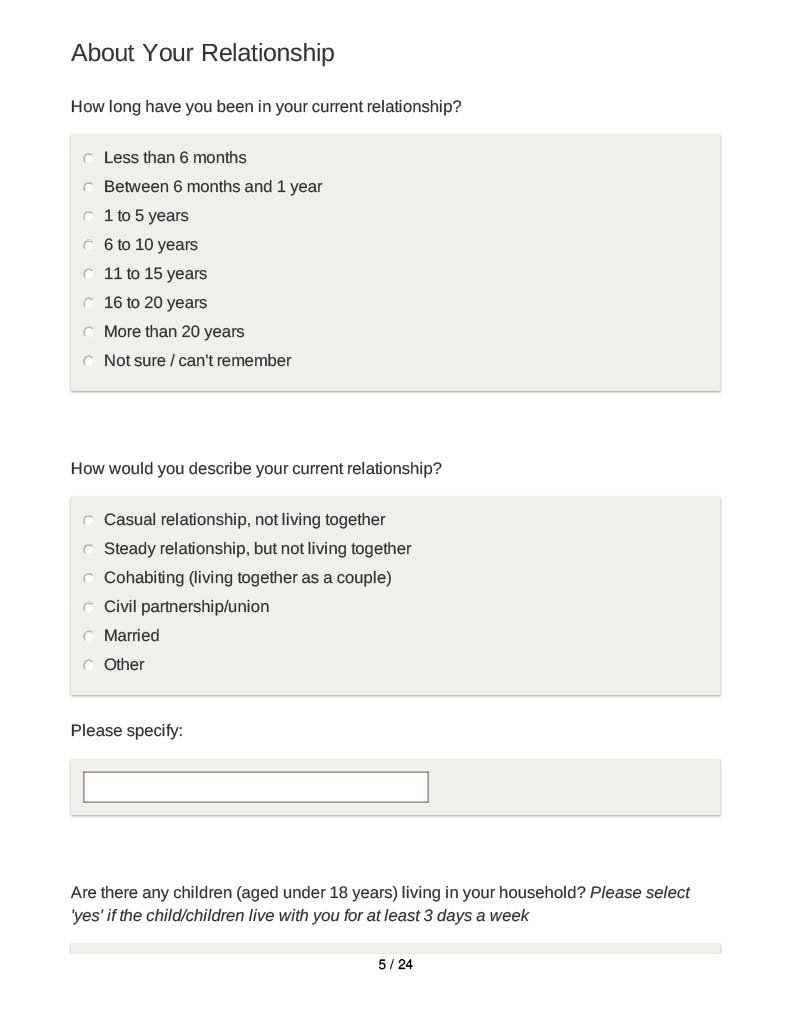

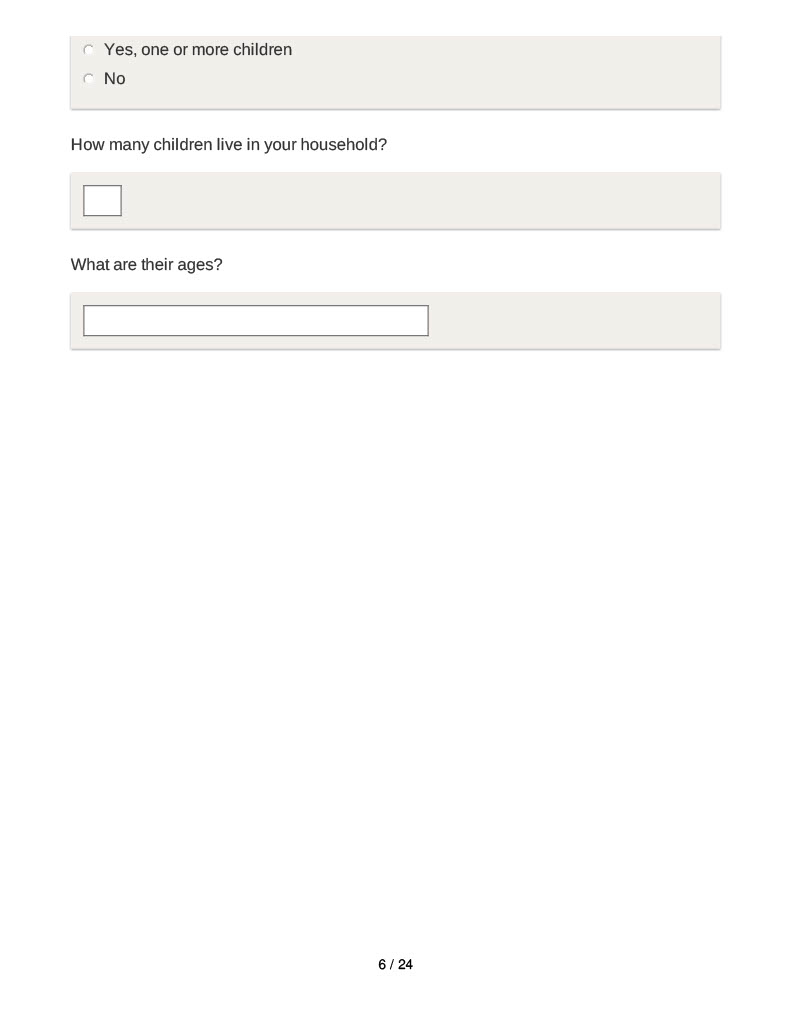

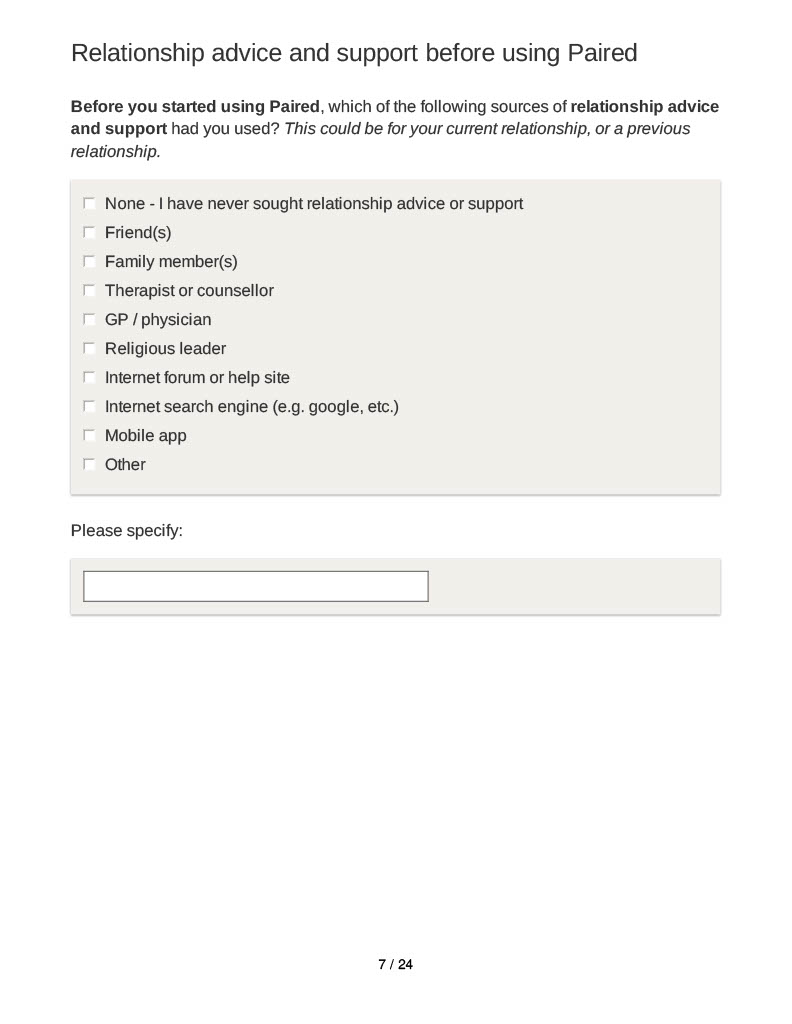

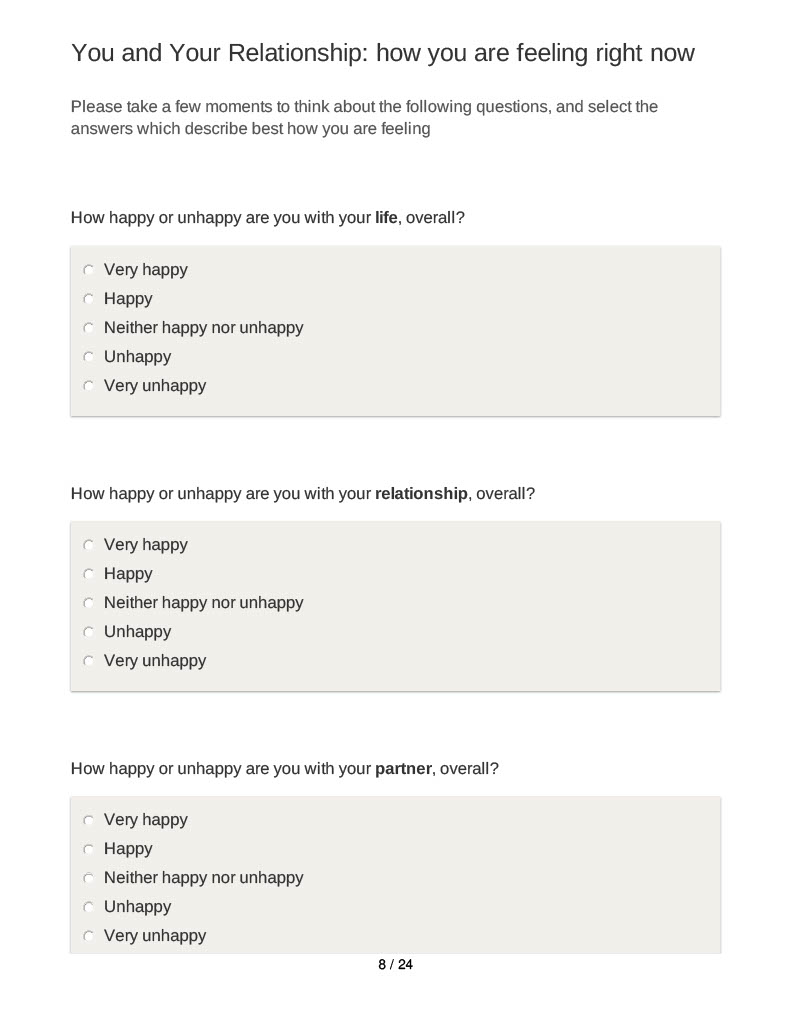

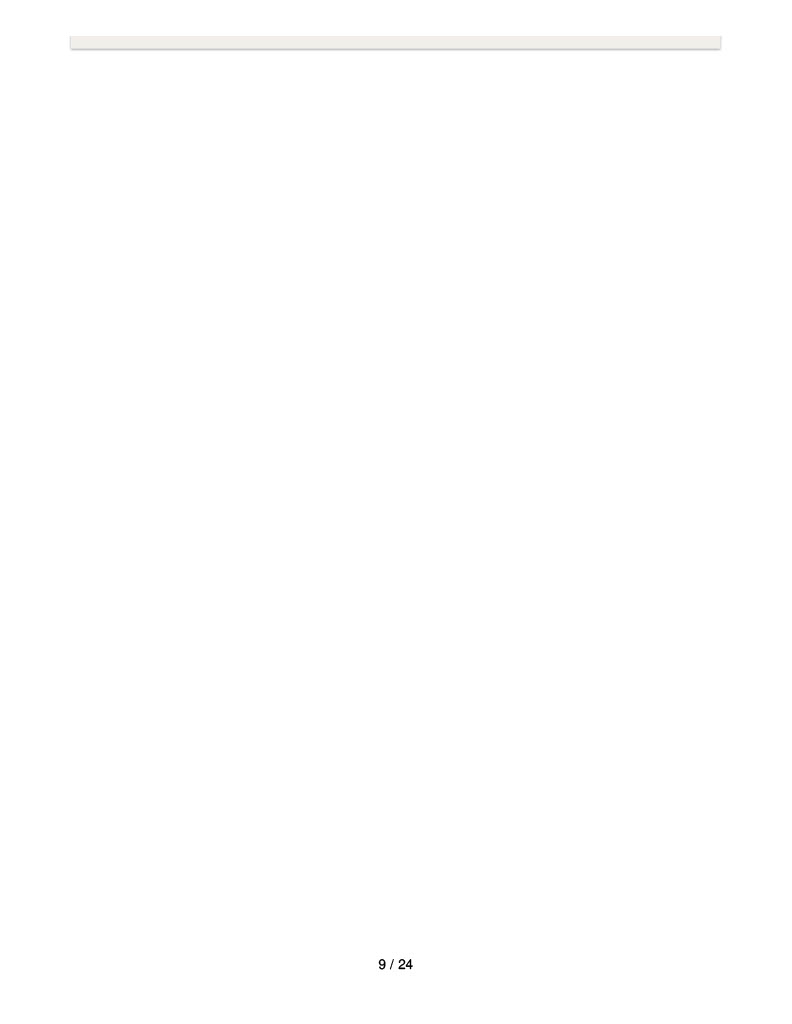

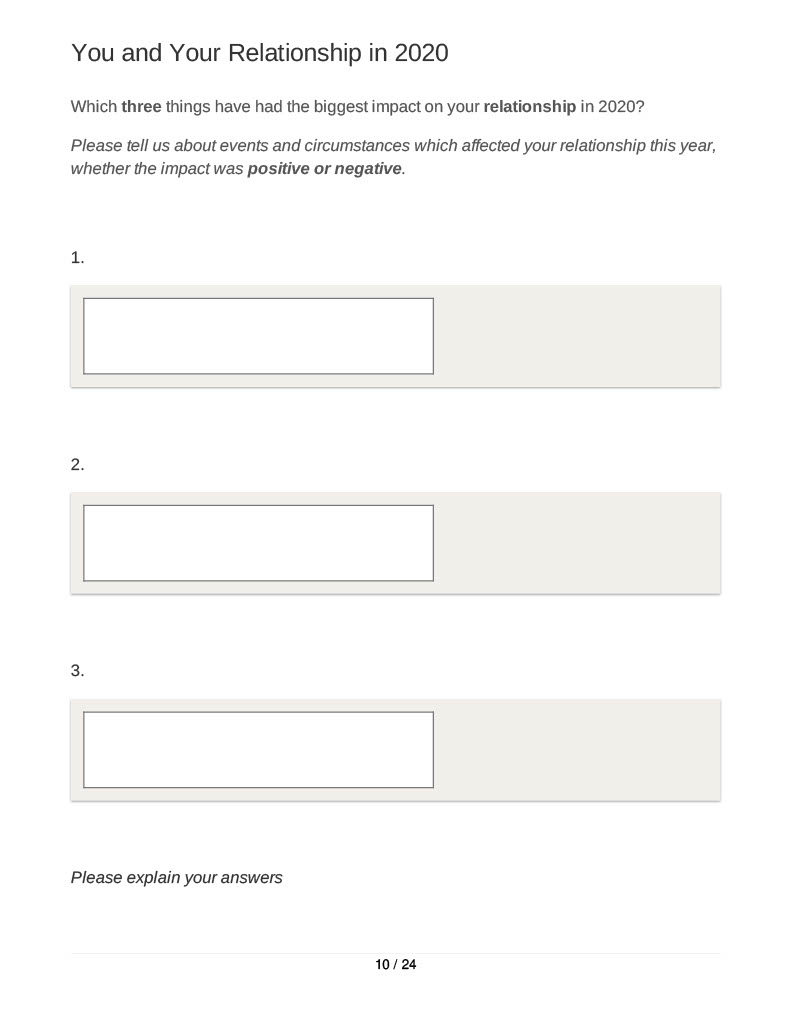

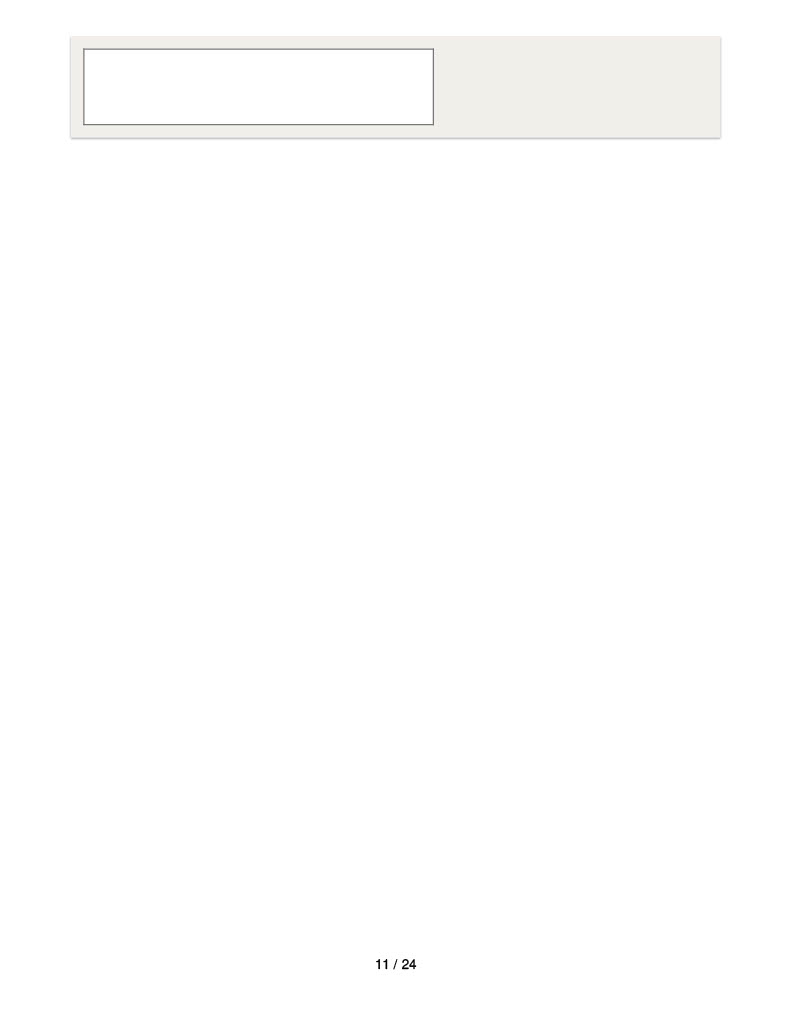

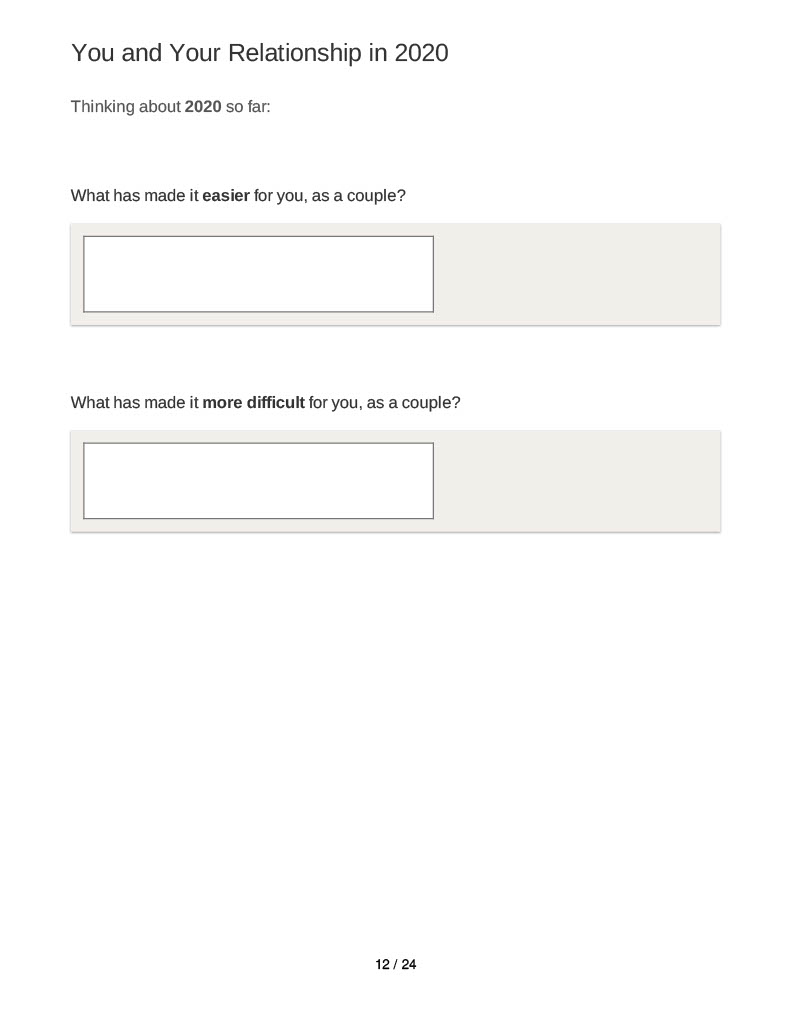

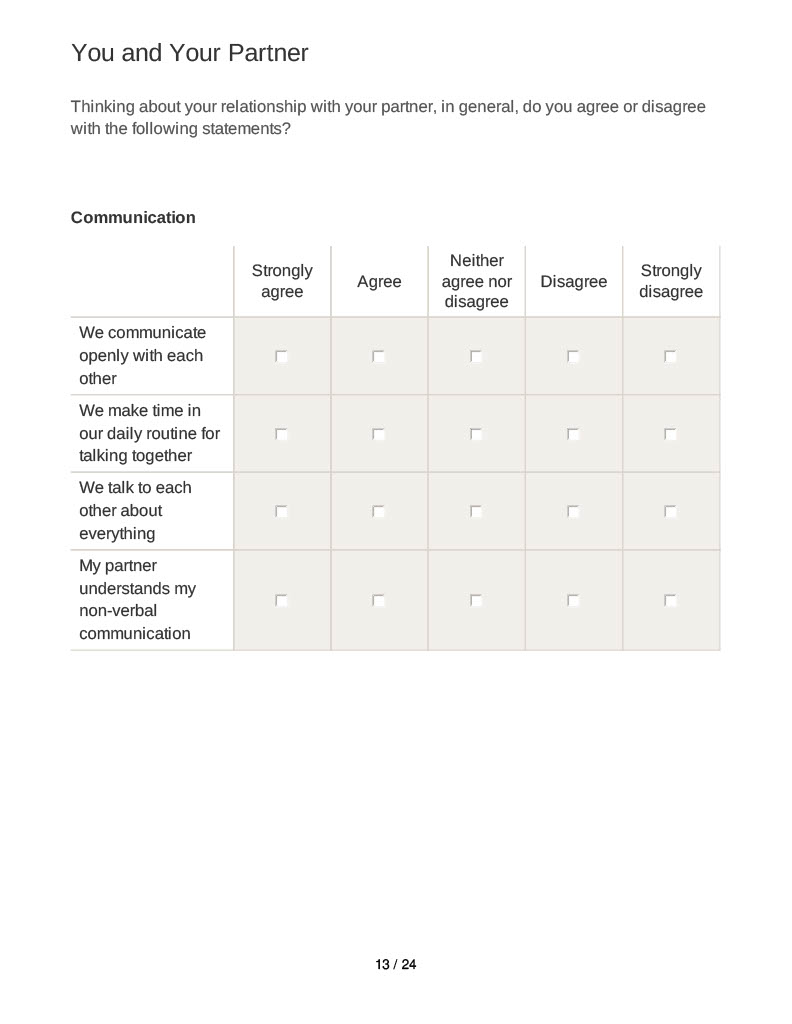

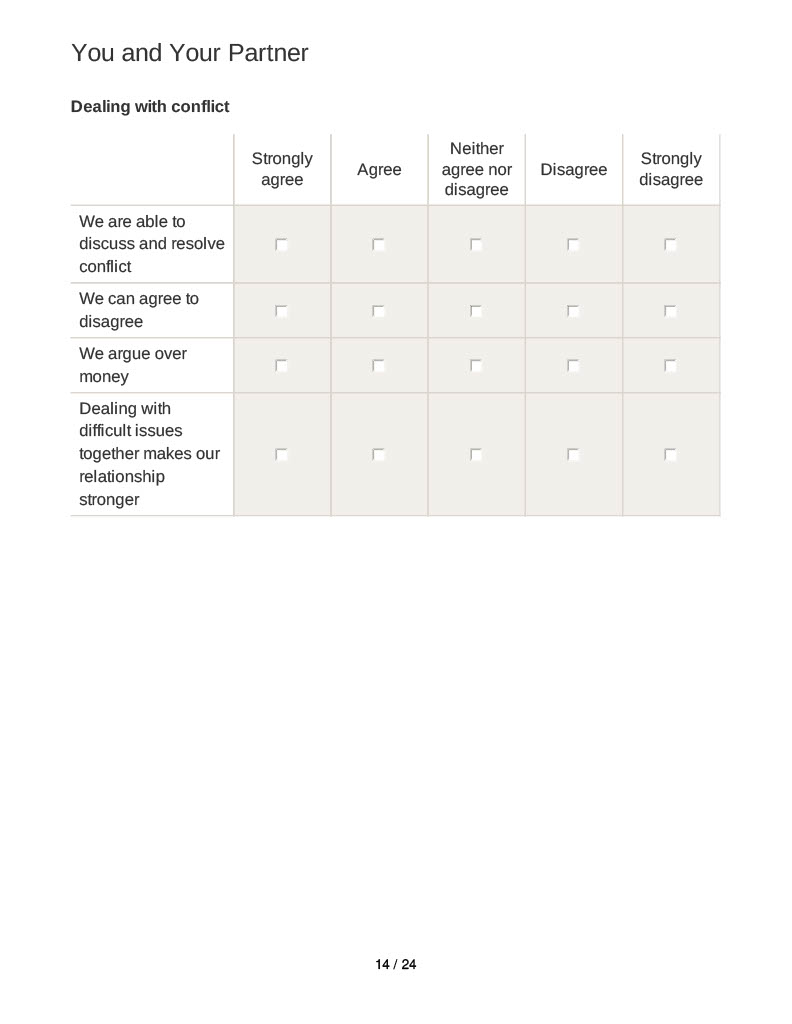

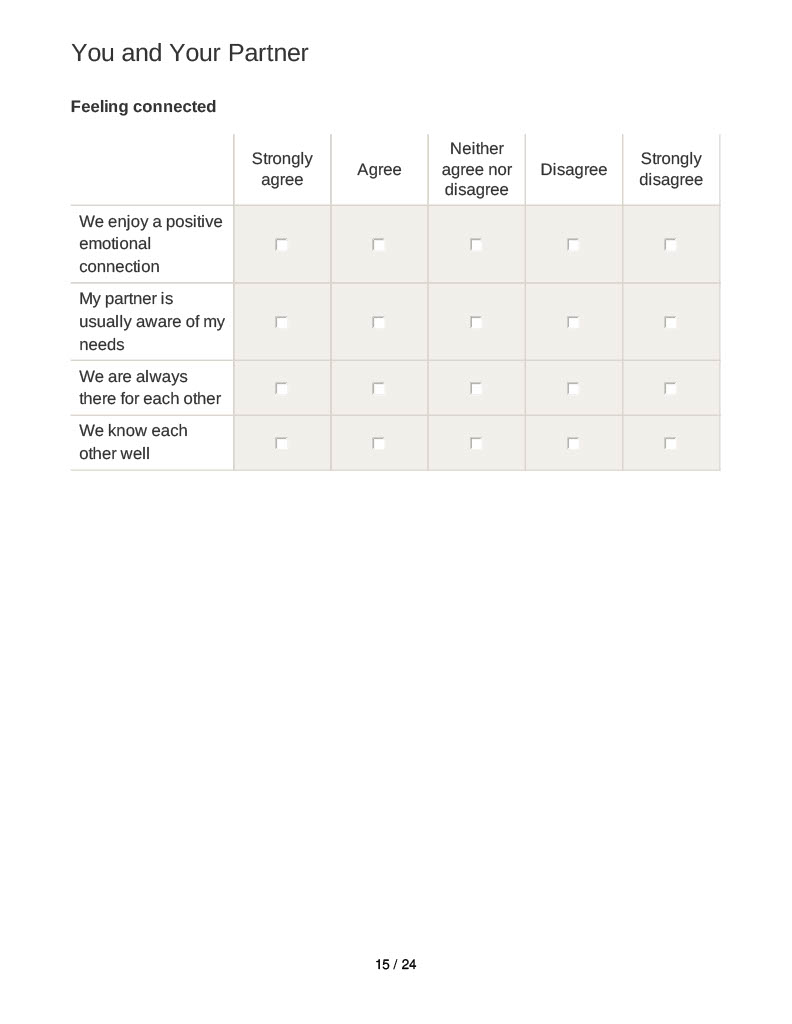

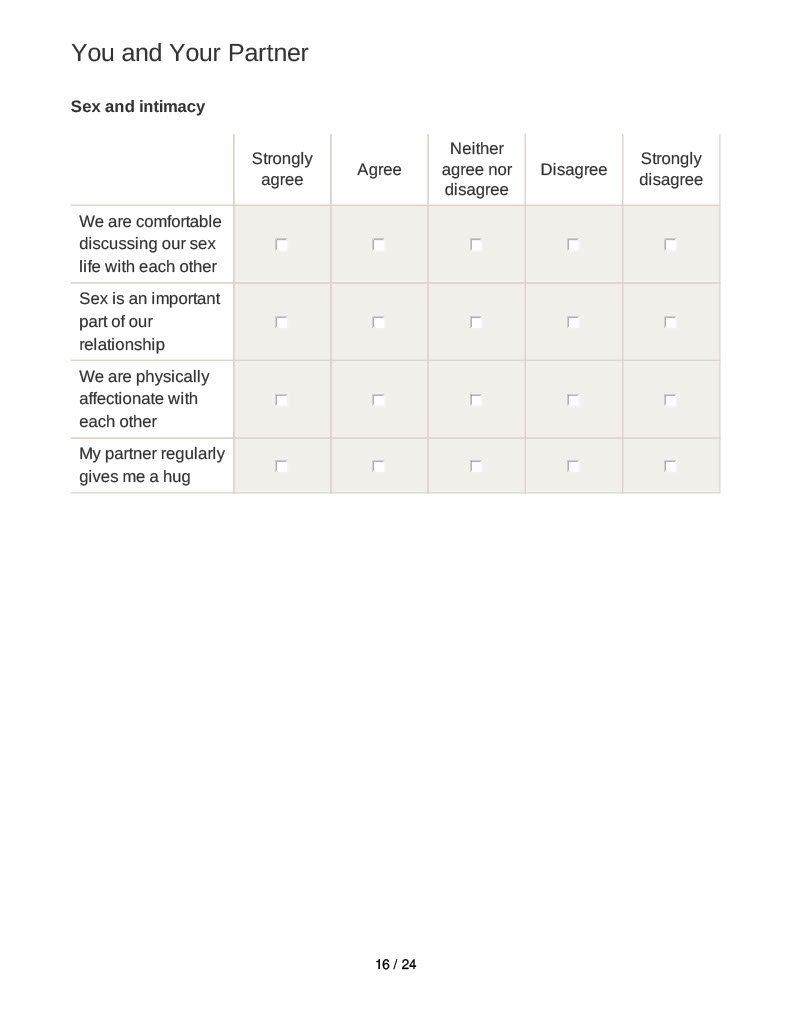

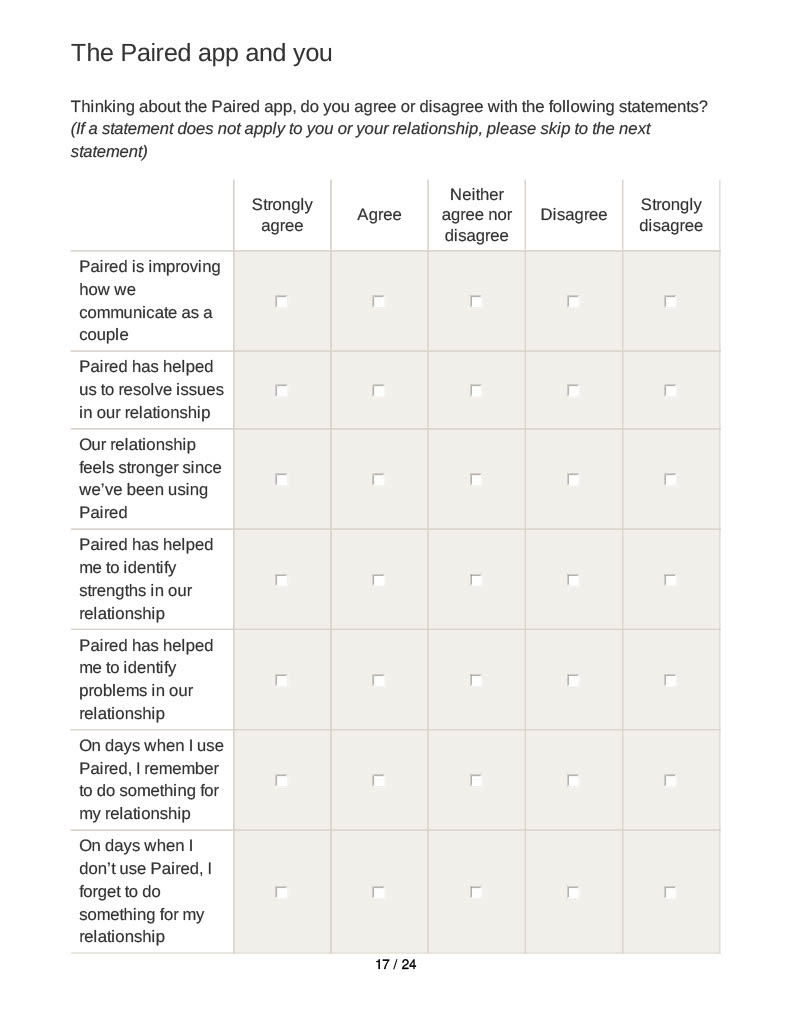

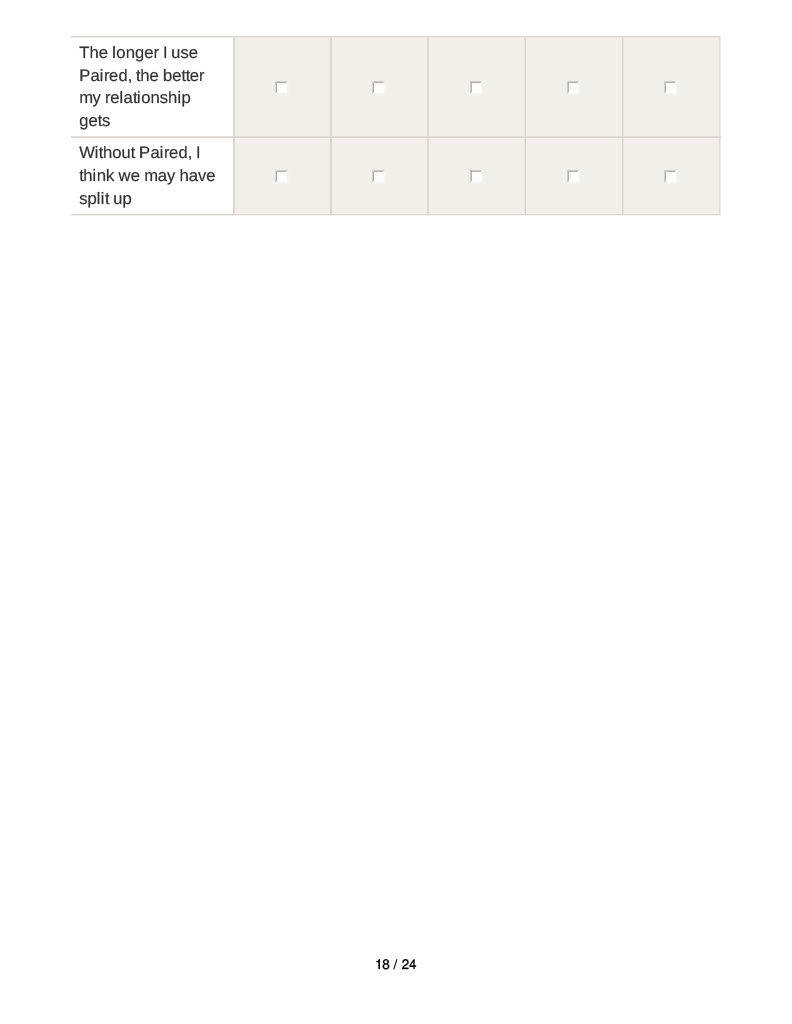

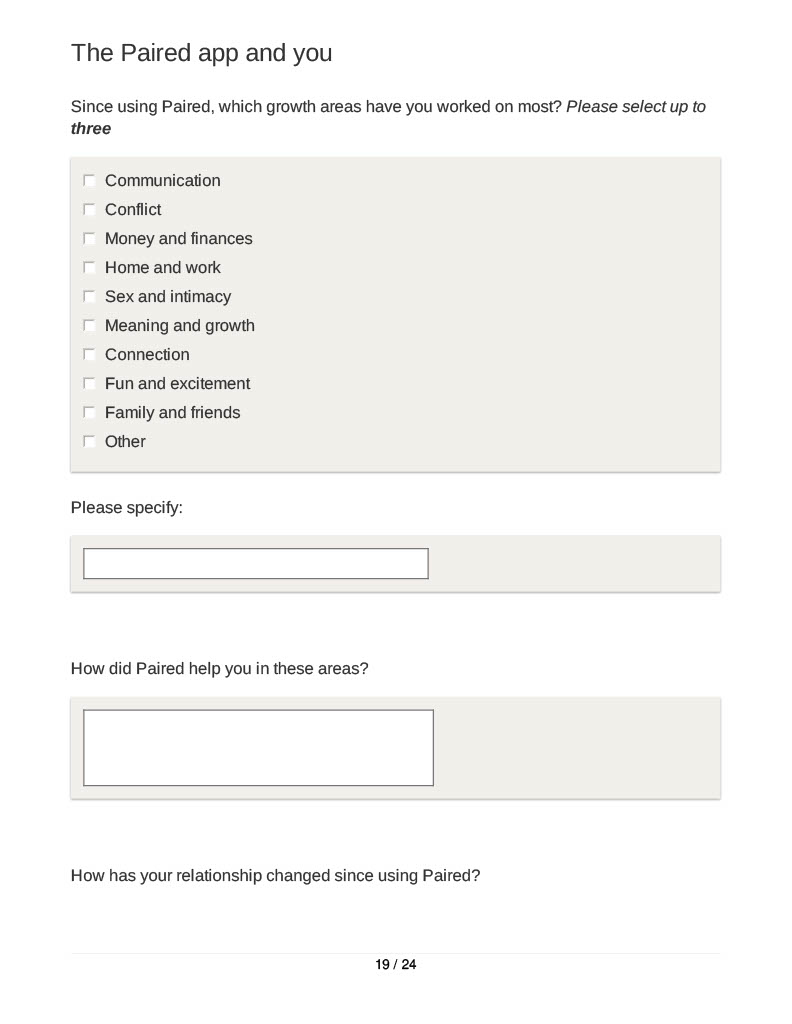

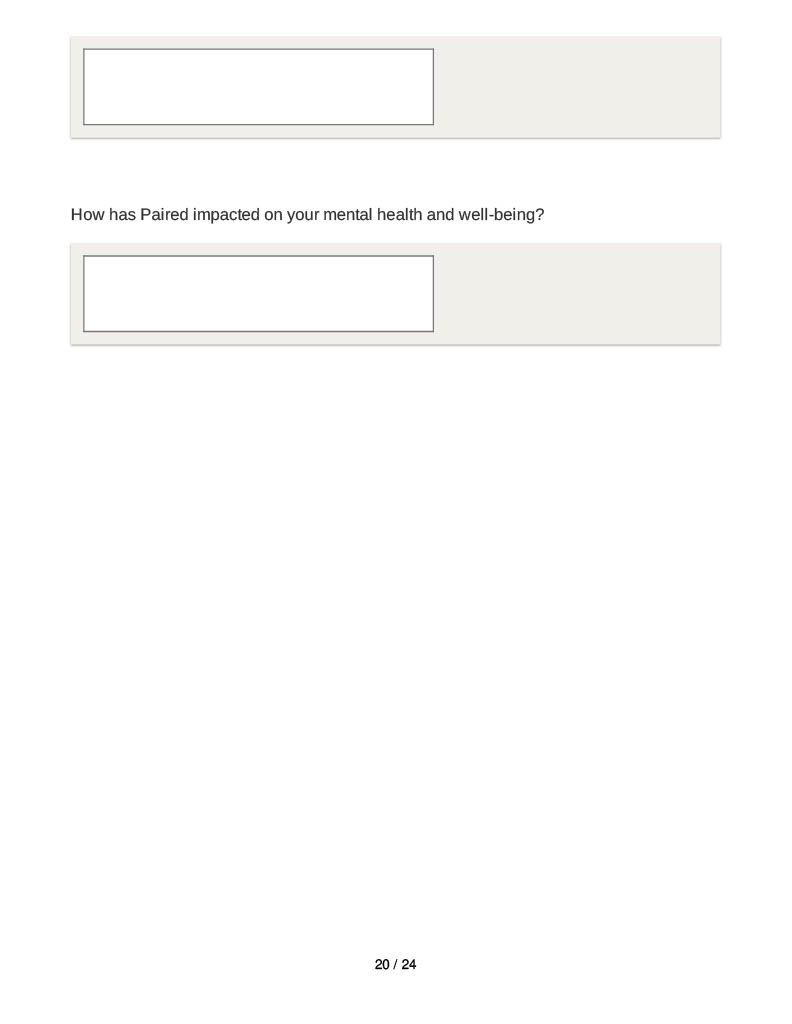

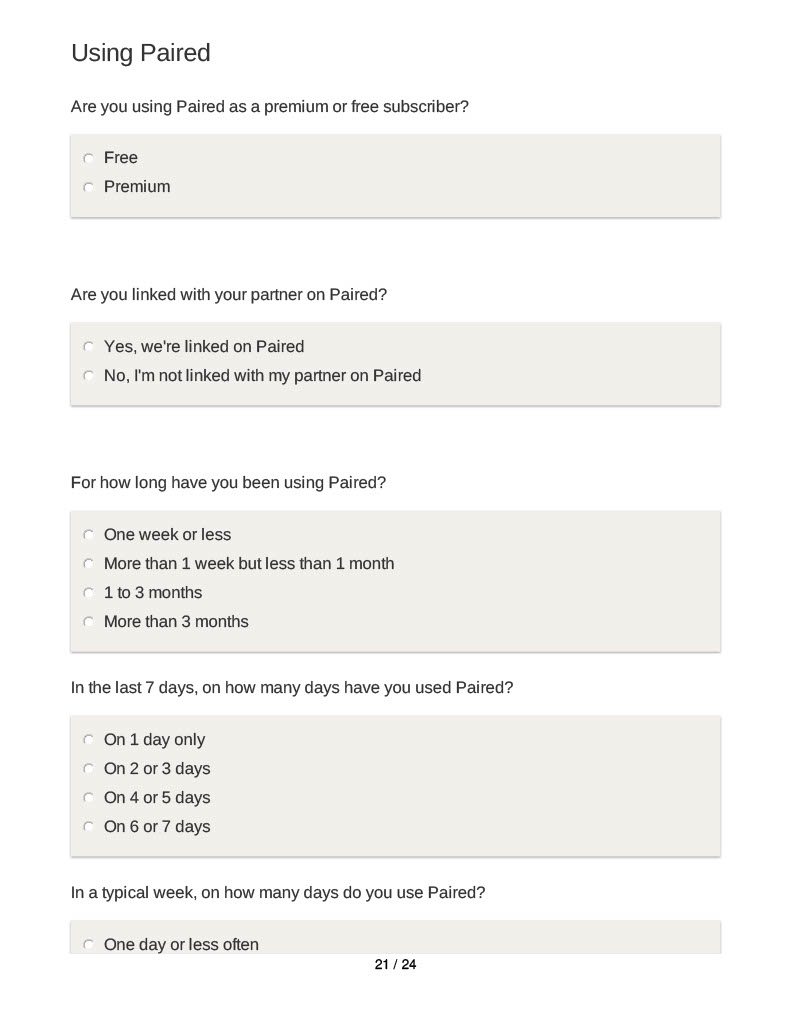

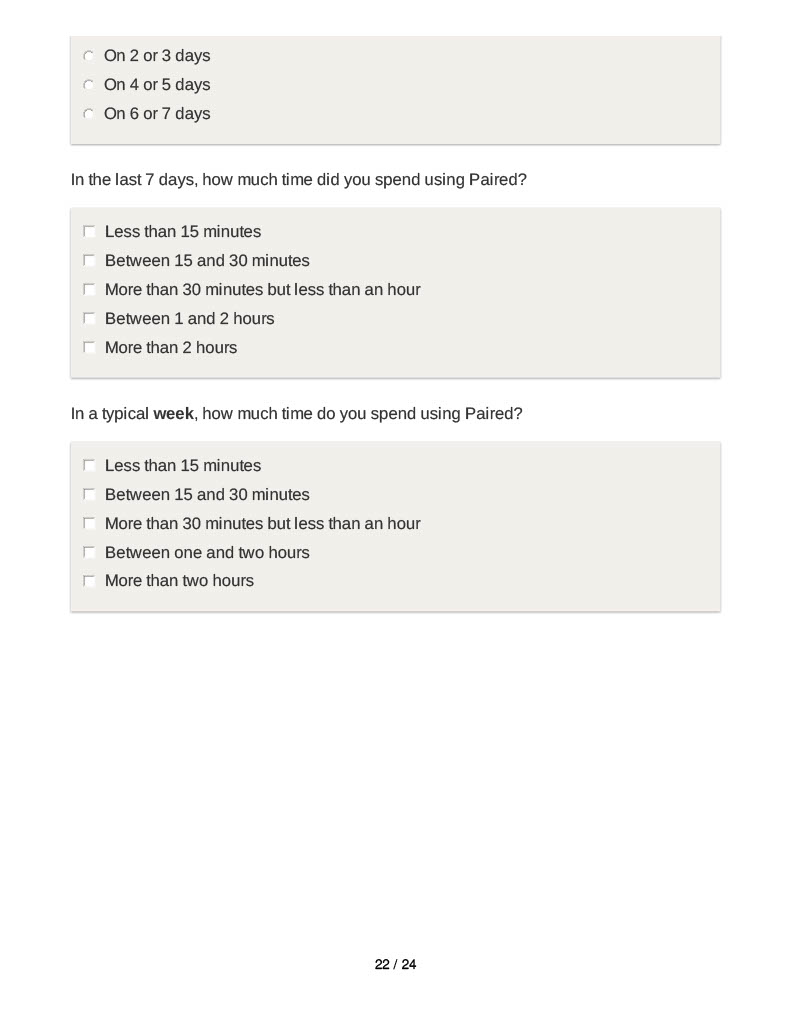

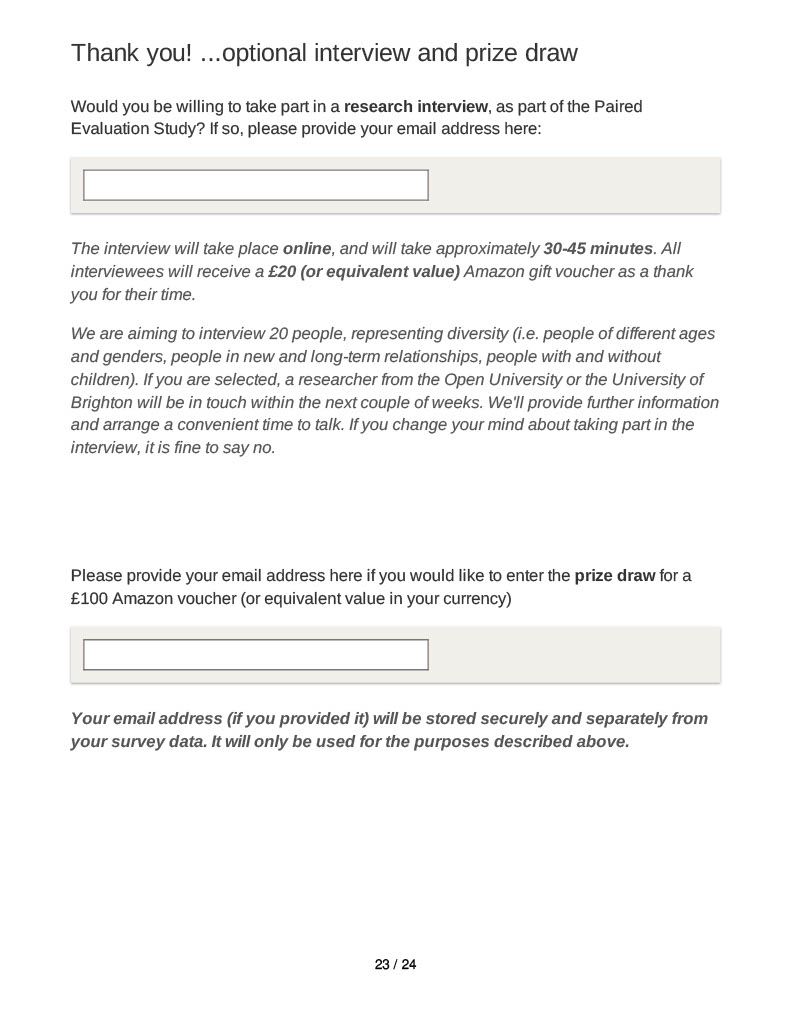

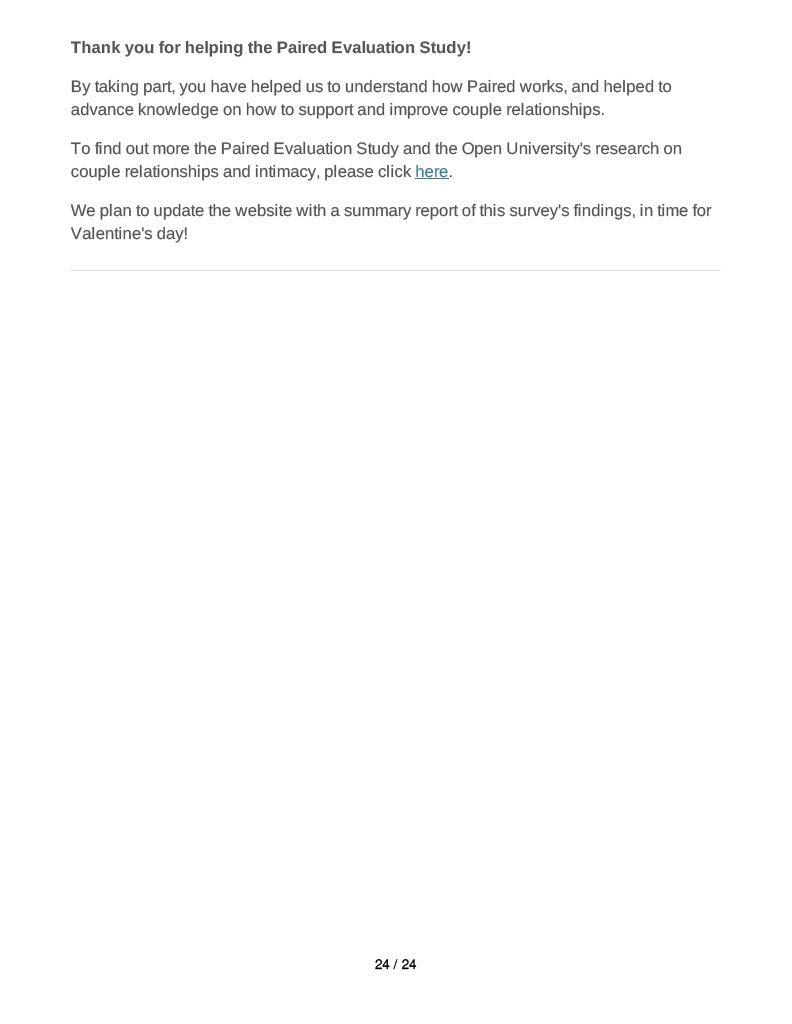


[MQoRS items are provided in Multimedia Appendix 2, and survey items]

### Topic guide for in-depth interviews (conducted online)

***Checklist***

• Check participant comfortable, quiet, secure location

• Check time available

• Agree procedure on breaks, connectivity issues

• Consent documented

• Reiterate anonymity – nothing you say to me will be linked back to you

• Final questions?

Informal interview, interested in your experiences... two broad sections (about you and your relationship, and then about *Paired* specifically)

**About you and your relationship**

- 1. • Tell me a bit about your relationship
     - 1. o Probes: Find out a little about the participant and their partner, their relationship type/duration, cohabiting or not, children? *[to cross check with survey info especially since some details may have changed since survey completion]*

*Relationship quality and wellbeing*

'Relationship quality' includes communication, connection, ability to manage conflict, and physical intimacy. In an everyday sense, these work as relationship maintenance behaviours that sustain the partnership

- 1. • How have the last few months been, for your relationship?
  2. o Probe: good things, bad things? Ambivalence?
  3. o Probe specifically about covid-related restrictions and other impacts (e.g. furlough, home-working, lockdown, quarantine if applicable)

• In terms of your own wellbeing, how have the last few months been?

o Probe: good things, bad things? Ambivalence?

o Probe specifically about covid-related restrictions and other impacts (e.g. furlough, home-working, lockdown if applicable)

Seeking support, advice and help with couple relationships

- Before using *Paired*, had you ever sought advice or help for any difficulties in relationships?
- Probe: for current relationship? For any previous relationships?
- Couple counselling / relationship counselling/therapy
- Lay help-seeking – e.g. via friends, family, religious institutions
- Websites, online forums, self-help books
- Other apps
- o Reasons? Type of help received? Experience?
- • And had you ever sought advice or help to support or improve the quality of your relationship?
  - o Probe: for current relationship? For any previous relationships?
- o From people (e.g. friends, family, counsellor)
- o From other sources (e.g. online, self-help books, apps)
- o Reasons? Type of help received? Experience?

Using *Paired*

• How/why did you start using *Paired*?

- - Probe: How long for?
  - Probe: Feelings about / reasons for engaging with an app vs. a person, for relationship support, circumstances of use; pros, cons
  - Probe: How do you feel when you use *Paired*? How has that changed over time?
  - Probe: Have you subscribed to the Premium? Can you talk me through how you decided that?
- What sort of changes have happened in your relationship since you started using *Paired*?
- Changes ascribed to *Paired* – gradual, instant, step-wise, not at all / not yet? Other circumstances & their impact on relationship
- Can you tell me more about how *Paired* has made a difference to your relationship? (positive or negative)
- Can you tell me about a time that *Paired* made a difference to your relationship? (positive or negative
- Probes:
- As a reminder to do regular ‘relationship work’
- As a way of facilitating/prompting communication with your partner
- As a way of fostering connection
- As a way of managing conflict
- As a way of enabling you and your partner to talk about sex and generate in a closer sense of intimacy
- Advice from relationship experts
- Content on specific topics – which?
- What do you like about it? Can you give me an example of what that means for you?
- Anything you are not so keen on? Can you give me an example of what that means for you?

‘Dailyness’

- How often do you use it? Can you talk me through how often you use it
- Any change over time, reason?
- Effectiveness

• Where and when do you tend to use it?

o Probes: Where are you, what else are you doing, routines and rituals?

Communication

- Has *Paired* changed how you communicate with your partner?
- How has *Paired* changed how you communicate with your partner?
- Can you tell me about time *Paired* made a difference to your communication?

*Paired* content and topics (‘Growth areas’)

- What content/features do you engage with? Why these?
- Which topics (‘growth areas’) have you looked at? Which do you find most helpful? In what ways?

Supporting the relationship

- Thinking broadly now, how (else) could *Paired* help your relationship?
  - Probe: Is there something else you’re intending to look at in the app, but have not yet had the chance?
- Is there new content/features that you would like to see in future?
  - Bear in mind they may mention something that *Paired* already deals with – e.g. something covered by a course (as not many people have engaged with this content, or they might mention something available only to premium users)

Sum up question

- Would you recommend *Paired* to others? If so, who would it best be suited to?
- What else would you like to tell me about *Paired*?

Thank you

Confirm email address to send voucher
