# Supplementary material for: Exploring the Potential of a Digital Intervention to Enhance Couple Relationships (the Paired App): Mixed Methods Evaluation
Source: JMIR Mhealth Uhealth. 2025 Apr 14;13:e55433. doi: 10.2196/55433 (PMC12001865; doi:10.2196/55433)
Supplement: Multimedia Appendix 2 [file mhealth_v13i1e55433_app2.docx]

## Multimedia Appendix 2: Multidimensional Quality of Relationship Scale (*MQoRS*) development, content, and analyses

### *MQoRS* development

The web-based survey included 16 statements addressing four aspects of relationship quality (including 4 statements matching those in the in-app quiz, and 4 statements matching those used in the *Enduring Love?* study (Gabb & Fink, 2015 [1]) and a further 2 statements about overall satisfaction with the relationship. Participants indicated agreement/disagreement on five-point Likert scales. The 18 statements were used to create the *MQoRS*, the final version of which comprises 17 statements across 5 domains. *MQoRS* scores were normalised to a range of 0-10 (low to high quality of relationship), to facilitate interpretation.

The *MQoRS* was generated through second order Confirmatory Factor Analysis (CFA) with Diagonally Weighted Least Squares estimator, as implemented in the lavaan package in R. The final model includes 17 congeneric variables, and provides adequate fit to the data: χ2(114) = 676.424, *P*<.001, CFI = 0.996, TLI = 0.995, RMSEA = 0.055, 90% CI [0.051, 0.059], SRMR = 0.041. High values of McDonald’s hierarchical omega coefficient (ω) and Average Variance Extracted (AVE), ranging from a minimum of ω = 0.75 and AVE = 0.59 for ‘Dealing with conflicts’, to a maximum of ω = 0.89 and AVE = 0.90 for ‘Happy with relationship’, indicate that the *MQoRS* reaches high levels of reliability and convergent validity. Similar results were found for the second-order factor, with a ω = 0.97 and AVE = 0.67. Standardised parameter estimates and inter-item reliability (R2) reach medium and mostly high levels, ranging from a minimum of β = 0.57 (R2 = 0.32) for ‘Sex is an important part of our relationship’, to a maximum of β = 0.97 (R2 = .94) for ‘How happy or unhappy are you with your relationship, overall?’.

Of the 18 items originally used to build the *MQoRS*, only one item ‘We argue over money’ was excluded due to a low factor score and inter-item reliability onto the corresponding factor ‘Dealing with conflicts’.

Further detail is provided in Di Martino et al, 2023 [2].

### *MQoRS* content

(Domains shown in bold, responses on 5-point Likert scales)

**Communication**

We communicate openly with each other

We make time in our daily routine for talking together

We talk to each other about everything

My partner understands my non-verbal communication

**Dealing with conflict**

We are able to discuss and resolve conflict

We can agree to disagree

Dealing with difficult issues together makes our relationship stronger

**Emotional connection**

We enjoy a positive emotional connection

My partner is usually aware of my needs

We are always there for each other

We know each other well

**Sex and intimacy**

We are comfortable discussing our sex life with each other

Sex is an important part of our relationship

We are physically affectionate with each other

My partner regularly gives me a hug

**Overall happiness with relationship**

How happy or unhappy are you with your relationship, overall?

How happy or unhappy are you with your partner, overall?

### R statistical software packages used: list

foreign = to read SPSS datasets (Team et al., 2020 [3])
lavaan = to implement CFA and Structural Equation Modelling (Rosseel, 2012 [4])
semTools = for additional statistics (e.g., Reliability and validity indexes)
ggplot2 = to build graphs (Wickham, 2011 [5])

ggstatsplot = to add statistical details to graphs (Patil, 2018 [6[)
dplyr = to create subgroups and filter variables (Wickham et al., 2015 [7])
ggrepel = to add labels to graphs (Slowikowski et al., 2016 [8])
car = to run statistical tests (e.g., ANOVA, t-test) (Fox et al., 2007 [9])
MKinfer = to run permutation t-tests (Kohl & Kohl, 2020 [10])
rstatix = for additional statistical tests (e.g., ANOVA with Welch approximation)
statsExpressions = to generate effect sizes (e.g., Cohen’s d, partial omega squared)

### Association between reported duration of use of *Paired* and *MQoRS* score: detail on statistical tests and results

**From the main text:** “Results from the web-based survey show that relationship quality, measured by the *MQoRS*, improved by 35.5% over 3 months’ *Paired* use, from 5.19 among new users, to 7.03 among people who had been using the app for more than 3 months (95%CIs: 44.7-53.3%, 31.1-43.7%, *P*<.01)”

**Detail:** Although equal variances were assumed (Levene test: F = 0.48, df (3,739), *P=*.69) due to the extremely unbalanced sample sizes, results of the differences between means are based on ANOVA with Welch approximation: F = 8.9, df (3, 55), *P*<.001. Based on partial omega-squared, a large effect size was detected for the omnibus test (ω2 = 0.29, 95% CI: 0.08, 0.45). A post hoc Tukey test showed statistically significant differences only between the group with the lowest score ‘One week or less’ and the group ‘1 to 3 months’ (∆x̄ = 1.50, *P=*.01, 95% CI: 0.24, 2.75), and ‘More than 3 months’, ∆x̄ = 1.83, *P=*.002, 95% CI: 0.52, 3.14). No statistically significant difference was found between the group with the highest mean score ‘More than 3 months’ and the group ‘1 to 3 months’ (∆x̄ = 0.85, *P*=.29, 95% CI: -0.82, 0.15).

### Association between reported frequency of use of *Paired* (number of days used in a typical week) and *MQoRS* score

**From the main text:** “Reporting more frequent *Paired* use was associated with a higher *MQoRS* score: people using *Paired* on 6-7 days per week scored 11.8% higher relationship quality than those using *Paired* on one day per week or less (*MQoRS* score 6.81 *vs.* 6.09) and this difference was statistically significant (*P*=.04, 95% CI for difference in *MQoRS*: 0.19, 1.42)”

**Detail:** Although equal variances were assumed (Levene test: F = 1.87, df (3,725), *P=*.13) due to the extremely unbalanced sample sizes, results of the differences between means are based on ANOVA with Welch approximation: F = 5.98, df (3, 149.68), *P*=.001. Based on partial omega-squared, a medium effect size was detected for the omnibus test (ω2 = 0.09, 95% CI: 0.01, 0.17). A post hoc Tukey test showed statistically significant differences only between the group with the highest score ‘On 6 or 7 days’ and the group who answered ‘On 2 or 3 days’ (∆x̄ = 0.60, *P=*.01, 95% CI: 0.11, 1.10), and the group with the lowest score ‘One day or less often’ (∆x̄ = 0.72, *P=*.04, 95% CI: 0.19, 0.42).

### Comparison of domains within the *MQoRS*

The five domains of the *MQoRS* are:

| **Domain** | **Strength of relationship to *MQoRS* score overall (maximum 10)** |
| --- | --- |
| Communication quality | 9.7 |
| Emotional connection | 9.5 |
| Capacity to deal with conflict as a couple | 7.9 |
| Sex and intimacy | 8.4 |
| Overall happiness with the relationship | 8.6 |

## References

1. Gabb J, Fink J. Couple Relationships in the 21st Century: Research, Policy, Practice. Cham, Switzerland. Springer; 2015. https://link.springer.com/book/10.1007/978-3-319-59698-3
2. Di Martino et al, 2023.
3. Team, R. C., Bivand, R., Carey, V. J., DebRoy, S., Eglen, S., Guha, R., ... & Pfaff, B. (2020). Package **‘foreign’**. Retrieved from: <https://cran.rapporter.net/web/packages/foreign/foreign.pdf>
4. Rosseel, Y. (2012). **Lavaan**: An R package for structural equation modeling and more. Version 0.5–12
   (BETA). Journal of statistical software, 48(2), 1-36.
5. Wickham, H. (2011). **ggplot2**. Wiley Interdisciplinary Reviews: Computational Statistics, 3(2), 180-185.
6. Patil, I. (2018). **ggstatsplot**: 'ggplot2' Based Plots with Statistical Details. Retrieved from: <https://cran.r-project.org/web/packages/ggstatsplot/index.html>
7. Wickham, H., Francois, R., Henry, L., & Müller, K. (2015). **dplyr**: A Grammar of Data Manipulation. R package version 0.4. 3. Retrieved from: <https://dplyr.tidyverse.org/>
8. Slowikowski, K., Schep, A., Hughes, S., Lukauskas, S., Irisson, J. O., Kamvar, Z. N., ... & Slowikowski, M. K. (2016). Package ‘**ggrepel’**. Retrieved from: <https://brieger.esalq.usp.br/CRAN/web/packages/ggrepel/ggrepel.pdf>
9. Fox, J., Friendly, G. G., Graves, S., Heiberger, R., Monette, G., Nilsson, H., ... & Suggests, M. A. S. S. (2007). The **car** package. R Foundation for Statistical Computing. Retrieved from <http://ftp.uni-bayreuth.de/math/statlib/R/CRAN/doc/packages/car.pdf>
10. Kohl, M., & Kohl, M. M. (2020). Package **‘MKinfer’**. Retrieved from: <https://cran.rstudio.org/web/packages/MKinfer/MKinfer.pdf>
