# Supplementary material for: Exploring the Potential of a Digital Intervention to Enhance Couple Relationships (the Paired App): Mixed Methods Evaluation
Source: JMIR Mhealth Uhealth. 2025 Apr 14;13:e55433. doi: 10.2196/55433 (PMC12001865; doi:10.2196/55433)
Supplement: Multimedia Appendix 3 [file mhealth_v13i1e55433_app3.docx]

## Multimedia Appendix 3: Additional statistical analyses

### Change in various aspects of relationship quality: further detail

#### Communication

At baseline, 13.4% of brief in-app survey participants strongly agreed that ‘I am very satisfied with how we communicate with each other’. Between the first and third brief survey, the proportion who strongly agreed with the statement ‘We communicate openly with each other’ rose from 47.0% to 59.9%. By three months’ use of *Paired*, 98% agreed or strongly agreed with this statement.

[Further details on the above: Based on multilevel multinomial logistic regression modelling with cumulative logit link function, a statistically significant fixed effect of duration of *Paired* use was observed on this ‘communication quality’ variable (F=106.41, df (3, 1753), *P*<.001) and variability in random intercepts (σ^2^=3.33, *P*<.001, 95% CI: 2.71, 4.10). There was a large statistically significant increase in the communication quality variable. Compared with baseline, an increase in this variable was observed with each successive brief survey (1^st^ brief survey: b=1.91, *P*<.001, 95% CI: 1.63, 2.20, OR=6.81, 95% CI: 5.14, 9.02; 2^nd^ brief survey: b=2.25, *P*<.001, 95% CI: 1.96, 2.54, OR=9.54, 95% CI: 7.14, 12.74; 3^rd^ brief survey: b=2.17, *P*<.001, 95% CI: 1.88, 2.45, OR=8.76, 95% CI: 6.57, 11.67). Predicted values were used to build Figure 2.]

Web-based survey data supported the observed benefits to communication: 76.1% participants (95%CI: 73.0-79.1%) agreed/strongly agreed with the statement ‘*Paired* is improving how we communicate as a couple’. This improvement was more commonly reported by those who had used *Paired* for one month or more, compared with newer users (80.5% *vs.* 66.1%, 95%CI: 77.1-83.9%, 59.9-72.3%, *P*<.001). Among web-based survey participants who had been using *Paired* for one month or more, we compared perceptions of *Paired*’s impact on couple communication by how many days they reported using the app in a typical week. Those who reported using *Paired* on 6 or 7 days per week were more likely to agree that it was improving their communication as a couple (84.9% vs. 74.0%; 95%CI: 81.0-88.8% and 67.9-80.1%, *P*=.002), compared with those who used it less often (5 days per week or fewer).

Taken together, findings support that *Paired* use is associated with improved couple communication quality. Reported communication quality increased over time (to three months) and with more frequent use. Improvements to couple communication are credited to *Paired*, particularly among people who have used the app for over a month, and daily.

#### Dealing with conflict

When they first used the app, around 16% of users reported that they strongly agreed with the statement ‘We are able to discuss and resolve conflict’. This almost doubled to 30% within the first month of using *Paired*; and tripled over the 3-month period to 46% (Figure 3). After 3 months, 93% agree or strongly agreed with the statement.

[Further details on the above: Based on multilevel multinomial logistic regression modelling with cumulative logit link function, a large statistically significant effect of duration of *Paired* use was observed on this ‘dealing with conflict’ variable (F=43.10, df (3, 1752), *P*<.001) and variability in random intercepts, (σ^2^= 4.31, *P*<.001, 95% CI: 3.54, 5.25). There was a large statistically significant increase in capacity to deal with conflict. Compared with baseline, an increase in this variable was observed with each successive brief survey (1^st^ brief survey: b=0.80, *P*<.001, 95% CI: 0.53, 1.07, OR=2.23, 95% CI: 1.71, 2.92; 2^nd^ brief survey: b=1.30, *P*<.001, 95% CI: 1.02, 1.57, OR=3.68, 95% CI: 2.79, 4.85; 3^rd^ brief survey: b=1.45, *P*<.001, 95% CI: 1.17, 1.73, OR=4.28, 95% CI: 3.24, 5.67). Predicted values were used to build Figure 3.]

This aspect of relationship quality was most closely measured in the web-based survey by a question asking the extent to which participants agreed that *Paired* had helped them to resolve issues within the relationship. Two out of five (39.8%) agreed or strongly agreed that *Paired* had helped them to resolve issues in their relationship (95%CI: 36.3-43.3%). This proportion was higher among people who had been using the app for longer (45.5% among people who had used *Paired* for one month or more, vs. 26.5% among those who had used it for a shorter period; 95%CI: 41.2-49.8% and 20.7-32.3%, *P*<.001 for difference). Among people who had used *Paired* for one month or more, those who used it on 6 or 7 days in a typical week were more likely to report this benefit compared to those who used it less often (49.5% vs. 39.3%; 95%CI: 44.0-55.0% and 32.5-46.1%, *P*=.02 for difference. Predicted values were used to build the corresponding Figure).

Taken together, findings support that *Paired* use is associated with improved capacity to deal with conflict. Reported capacity to deal with conflict increased over time (to three months) and with more frequent use of the app. *Paired* users were more likely to credit the app with helping them resolve issues in their relationships if they had been using the app for over a month, and daily.

#### Emotional connection

When they first started using the app, *Paired* users were asked the extent to which they agreed or disagreed with the statement ‘I feel connected with my partner emotionally’, and in the brief survey the corresponding statement was ‘We enjoy a positive emotional connection’. At the outset, a high 98% agreed or strongly agreed that they felt emotionally connected with their partner, which leaves little room for improvement. Nevertheless, the proportion selecting ‘strongly agree’ rose from around three-in-five to almost four-in-five (61.4% to 77.5%).

[Further detail: Based on multilevel multinomial logistic regression modelling with cumulative logit link function, a moderate statistically significant fixed effect of duration of *Paired* use was observed on this dealing with conflict variable (F=17.41, df (3, 1752), *P*<.001) and variability in random intercepts, (σ^2^=3.44, *P*<.001, 95% CI: 2.79, 4.23). Compared with baseline, an increase in this variable was observed with each successive brief survey (1^st^ brief survey: b=0.75, *P*<.001, 95% CI: 0.45, 1.06, OR=2.13, 95% CI: 1.57, 2.89; 2^nd^ brief survey: b=0.98, *P*<.001, 95% CI: 0.67, 1.29, OR=2.67, 95% CI: 1.95, 3.66; 3^rd^ brief survey: b=0.93, *P*<.001, 95% CI: 0.62, 1.24, OR=2.55, 95% CI: 1.86, 3.48). Predicted values were used to build Figure 4.]

#### Sex and intimacy

We found a small increase in how comfortable people reported feeling about discussing their sex life. At the outset, 44.8% of people strongly agreed that they felt comfortable with this, rising to 54.5% by 3 months (Figure 5). This is an aspect of relationship quality which may be less affected by short-term use of *Paired* (especially compared with communication, or capacity to deal with conflict), and where many *Paired* users started from relatively high level of reported openness, considering these matters are sensitive to discuss.

[Further detail: Results from multilevel multinomial logistic regression models with cumulative logit link function reveal a statistically significant fixed effect of duration of *Paired* use was found on the variable corresponding to the statement ‘We are comfortable discussing our sexual life’ (F = 5.69, df (3, 1752), *P=*.001) and variability in random intercepts, (σ2 = 6.41, *P*<.001, 95% CI: 5.30, 7.75). Compared with baseline, a small increase in this variable was observed with each successive brief survey (1st brief survey: b = 0.39, *P=*.01, 95% CI: 0.10, 0.68, OR = 1.48, 95% CI: 1.11, 1.97; 2nd brief survey: b = 0.48, *P=*.001, 95% CI: 0.19, 0.77, OR = 1.62, 95% CI: 1.21, 2.16; 3^rd^ brief survey: b = 0.55, *P*<.001, 95% CI: 0.26, 0.84, OR = 1.74, 95% CI: 1.30, 2.33). Predicted values were used to build the Figure 5.]

### Comparison of perceived effectiveness of *Paired* by demographic characteristics

**Table S1: Perceived effectiveness of *Paired* by demographic characteristics, among web-based survey participants who reported having used *Paired* for at least one month**

Chi-squared test, logistic regression to obtain odds ratios.

The rationale for restricting this analysis to survey participants who reported at least one month’s use of *Paired* is to focus on those who have used the app for a reasonable time period. Restriction to people who have used the app for at least a month also reduces the potentially confounding impact of differences in duration of *Paired* app use by demographics (which might occur due to, eg, media attention or promotion targeting a particular group shortly before web-based survey data collection). If very new users of the app were overrepresented in one demographic group and underrepresented in another, it would be misleading to compare the effectiveness of *Paired* between the two groups.

|  | n | ***Paired* is improving how we communicate as a couple** | | | **Our relationship feels stronger since we’ve been using *Paired*** | | | **The longer I use *Paired*, the better my relationship gets** | | |
| --- | --- | --- | --- | --- | --- | --- | --- | --- | --- | --- |
|  |  | % agree or  strongly agree | OR (95% CI) | p | % agree or  strongly agree | OR (95% CI) | p | % agree or  strongly agree | p, OR (95% CI) |  |
| **All** | **514** | **80.5%** |  |  | **64.3%** |  |  | **49.0%** |  |  |
|  |  |  |  |  |  |  |  |  |  |  |
| Gender |  |  |  | 0.549* |  |  | 0.548* |  |  | 0.968* |
| Female | 352 | 81.3% | 1.0 |  | 65.1% | 1.0 |  | 49.1% | 1.0 |  |
| Male | 152 | 78.9% | 0.87 (0.54-1.39) |  | 62.3% | 0.87 (0.59-1.32) |  | 49.3% | 1.01 (0.69-1.47) |  |
| Other | 9 | 77.8% | * |  | 77.8% | * |  | 44.4% | * |  |
|  |  |  |  |  |  |  |  |  |  |  |
| Age |  |  |  | 0.380 |  |  | 0.955 |  |  | 0.877 |
| 18-24 | 212 | 80.7% | 1.0 |  | 63.2% | 1.0 |  | 50.5% | 1.0 |  |
| 25-34 | 176 | 83.5% | 1.22 (0.72-2.05) |  | 64.6% | 1.06 (0.70-1.61) |  | 48.3% | 0.92 (0.62-1.37) |  |
| 35-44 | 77 | 74.0% | 0.68 (0.37-1.26) |  | 64.9% | 1.08 (0.63-1.86) |  | 45.5% | 0.82 (0.49-1.38) |  |
| 45+ | 49 | 79.6% | 0.94 (0.43-2.03) |  | 67.3% | 1.20 (0.62-2.32) |  | 51.0% | 1.02 (0.55-1.90) |  |
|  |  |  |  |  |  |  |  |  |  |  |
| Sexual orientation |  |  |  | 0.657 |  |  | 0.943 |  |  | 0.811 |
| Heterosexual | 377 | 81.2% | 1.0 |  | 64.4% | 1.0 |  | 48.8% | 1.0 |  |
| LGBTQ+ | 136 | 79.4% | 0.95 (0.74-1.21) |  | 64.7% | 1.01 (0.82-1.24) |  | 50.0% | 1.02 (0.84-1.25) |  |
|  |  |  |  |  |  |  |  |  |  |  |
| Child(ren) aged <18 living in household |  |  |  | 0.236 |  |  | 0.048 |  |  | 0.336 |
| Yes | 145 | 77.2% | 1.0 |  | 57.6% | 1.0 |  | 52.4% | 1.0 |  |
| No | 369 | 81.8% | 1.33 (0.83-2.13) |  | 66.9% | 1.49 (1.00-2.21) |  | 47.7% | 0.83 (0.56-1.21) |  |
|  |  |  |  |  |  |  |  |  |  |  |
| Country |  |  |  | 0.236 |  |  | 0.275 |  |  | 0.688 |
| UK | 219 | 81.3% | 1.0 |  | 61.6% | 1.0 |  | 47.5% | 1.0 |  |
| US | 204 | 78.9% | 1.16 (0.72-1.87) |  | 68.6% | 0.74 (0.49-1.10) |  | 51.5% | 0.85 (0.58-1.25) |  |
| Other | 90 | 82.2% | 0.94 (0.50-1.78) |  | 61.8% | 0.99 (0.60-1.65) |  | 47.8% | 0.99 (0.61-1.62) |  |
|  |  |  |  |  |  |  |  |  |  |  |
| Relationship type |  |  |  | 0.839* |  |  | 0.541* |  |  | 0.369* |
| Not cohabiting | 191 | 79.1% | 1.0 |  | 61.8% | 1.0 |  | 48.2% | 1.0 |  |
| Cohabiting | 150 | 81.3% | 1.15 (0.67-1.98) |  | 67.3% | 1.28 (0.81-2.00) |  | 46.0% | 0.92 (0.60-1.41) |  |
| Married/partnered | 164 | 81.1% | 1.14 (0.67-1.92) |  | 65.6% | 1.18 (0.77-1.83) |  | 53.7% | 1.25 (0.82-1.89) |  |
| Other | 9 | 88.9% | * |  | 44.4% | * |  | 33.3% | * |  |
|  |  |  |  |  |  |  |  |  |  |  |
| Relationship duration |  |  |  | 0.880 |  |  | 0.423 |  |  | 0.185 |
| =< 1 year | 109 | 81.7% | 1.0 |  | 67.0% | 1.0 |  | 53.2% | 1.0 |  |
| 1-5 years | 266 | 79.7% | 0.88 (0.50-1.56) |  | 61.7% | 0.79 (0.50-1.27) |  | 45.1% | 0.72 (0.46-1.13) |  |
| >5 years | 139 | 81.3% | 0.98 (0.51-1.86) |  | 67.4% | 1.02 (0.60-1.74) |  | 53.2% | 1.00 (0.61-1.67) |  |

*Due to very small numbers in one response category for the variables *gender* and *relationship type* (gender: other; relationship type: other), these response categories were not included in statistical tests. Therefore no odds ratios are presented. The p-values for comparisons by *gender* refer to comparisons between females and males. The p-values for comparisons by *relationship type* refer to comparisons between participants who reported not cohabiting with their partner; participants who reported cohabiting; and participants who reported being married or civil partnered. We present percentages for people of other genders, and people in other relationship types, for completeness, but we urge caution in interpreting these because the numbers are small (n<10).
