# Supplementary material for: Exploring the Potential of a Digital Intervention to Enhance Couple Relationships (the Paired App): Mixed Methods Evaluation
Source: JMIR Mhealth Uhealth. 2025 Apr 14;13:e55433. doi: 10.2196/55433 (PMC12001865; doi:10.2196/55433)
Supplement: Multimedia Appendix 4 [file mhealth_v13i1e55433_app4.docx]

## Multimedia appendix 4: Comparison of web-based survey participants and *Paired* users

This comparison is limited by differences between the demographic data collected by *Paired*, and that collected by our web-based survey. *Paired* users’ demographics and relationship characteristics were estimated based on aggregate data on the 5468 *Paired* users who had volunteered these details to the app by early January 2021; partner linkage data and country of residence data was based on all ‘active users’ of the app as defined by *Paired*, in January 2021.

|  |  | Web-based survey participants | *Paired* users  (data from *Paired*) |
| --- | --- | --- | --- |
| **Demographics** | | | |
| Age, years | *Mean (SD)* | *29.7 (9.6)* | *29.6 (9.1)* |
| Gender | Female | 67.1% | 62.8% |
|  | Male | 31.5% | 35.0% |
|  | Other gender identities | 1.3% | 2.3% |
| Sexual orientation | Heterosexual | 74.3% | Not asked |
|  | LGBT+, *of which:* | 25.7% | Not asked |
|  | *Gay/lesbian* | *5.1%* | Not asked |
|  | *Bisexual* | *18.1%* | Not asked |
|  | *Other sexualities* | *2.4%* | Not asked |
| Children?^a^ | Yes | 29.7% | 38.7% |
|  | No | 70.3% | 58.7% |
|  | Expecting | Not asked | 2.6% |
| Country of residence | USA | 44.8% | Most *Paired* users resided in US or UK |
|  | UK | 34.9% |  |
|  | Other countries | 20.3% |  |
| **Relationship characteristics**  *Web-based survey and Paired data are not directly comparable so are presented in different rows* | | | |
| Relationship status^b^ | Casual relationship, not living together | 1.5% | - |
|  | Steady relationship, not living together | 33.0% | - |
|  | Cohabiting (living together as a couple) | 30.7% | - |
|  | Other | 1.5% | - |
|  | Civil partnership/union | 1.3% | - |
|  | Married | 32.0% | - |
|  | Other | 1.5% | - |
| Living with partner? | Yes | - | 66.4% |
|  | No | - | 33.6% |
| Marital status | Married | - | 29.4% |
|  | Civil partnership | - | 9.4% |
|  | Unmarried | - | 61.3% |
| Relationship duration | >6 months | 4.7% | - |
|  | 6 months to 1 year | 15.6% | - |
|  | 1 to 5 years | 50.1% | - |
|  | 6 to 10 years | 15.8% | - |
|  | 11 to 15 years | 7.7% | - |
|  | 16 to 20 years | 3.4% | - |
|  | More than 20 years | 2.8% | - |
|  | ‘Average’ year started | - | 2015/2016 |
|  | Range | - | 1965-2021 |
|  | Standard deviation | - | 6.0 years |
| ***Paired* usage** | | | |
| Subscription | Free | 80.8% | ^d^ |
|  | Premium | 19.2% | ^d^ |
| Linked with partner, on *Paired*? | Yes | 89.9% | 85-90%^e^ |
|  | No | 10.1% | 10-15%^e^ |

^a^Web-based survey participants were asked whether they had children aged under 18 living in their household, and were not provided with the response option ‘expecting’. In contrast, *Paired* users were asked whether they had children and the question did not specify children’s ages or whether they were living with them. ^b^The web-based survey asked one question about relationship status; *Paired* asked two separate questions about marital status and cohabitation. ^c^Web-based survey participants were provided with seven response options to choose from, as shown. *Paired* subscribers were asked to indicate the year in which their relationship started. ^d^Commercially sensitive; data not provided by *Paired*. ^e^Estimated based on the proportion of active users of *Paired* who are linked with a partner via the app.
